# The evolution of reproductive modes and life cycles in amphibians

**DOI:** 10.1038/s41467-022-34474-4

**Published:** 2022-11-17

**Authors:** H. Christoph Liedtke, John J. Wiens, Ivan Gomez-Mestre

**Affiliations:** 1 Ecology, Evolution and Development Group, Doñana Biological Station (CSIC), 41092 Sevilla, Spain; 2 Department of Ecology and Evolutionary Biology, University of Arizona, Tucson, AZ 85721-0081 USA

**Keywords:** Phylogenetics, Biodiversity, Herpetology

## Abstract

Amphibians have undergone important evolutionary transitions in reproductive modes and life-cycles. We compare large-scale macroevolutionary patterns in these transitions across the three major amphibian clades: frogs, salamanders, and caecilians. We analyse matching reproductive and phylogenetic data for 4025 species. We find that having aquatic larvae is ancestral for all three groups and is retained by many extant species (33–44%). The most frequent transitions in each group are to relatively uncommon states: live-bearing in caecilians, paedomorphosis in salamanders, and semi-terrestriality in frogs. All three groups show transitions to more terrestrial reproductive modes, but only in caecilians have these evolved sequentially from most-to-least aquatic. Diversification rates are largely independent of reproductive modes. However, in salamanders direct development accelerates diversification whereas paedomorphosis decreases it. Overall, we find a widespread retention of ancestral modes, decoupling of trait transition rates from patterns of species richness, and the general independence of reproductive modes and diversification.

## Abstract (web summary)

Here, the authors use reproductive mode data with matching phylogenetic data to explore the evolution of reproductive mode, transitions between reproductive modes, and diversification rates in amphibians.

## Introduction

The majority of animals have complex life cycles^1^, consisting of one or more larval phases and an adult phase (typically with distinct morphologies and ecologies), separated by extensive remodeling of body plans (metamorphosis). However, major clades (e.g., amniotes), have lost the larval stage and exhibit a uniphasic life cycle^2^. How this loss comes about is an important evolutionary question. This loss has occurred frequently in non-amniote vertebrates, especially Amphibia. Since their origin ~300 million years ago^3^, amphibians have evolved many alternatives to their ancestral biphasic life cycle^4,5^, including loss of larval stages (through direct development or viviparity) and loss of the adult stage (paedomorphosis). Here we use a phylogenetic approach to test hypotheses about the evolution of reproductive modes and life cycles among the three major amphibian clades (frogs [Anura], salamanders [Caudata], and caecilians [Gymnophiona]) and their possible consequences for lineage diversification rates. More broadly, we evaluate whether large-scale patterns of reproductive-mode and life-cycle evolution are similar or different among major clades (and how).

**Fig. 1:**
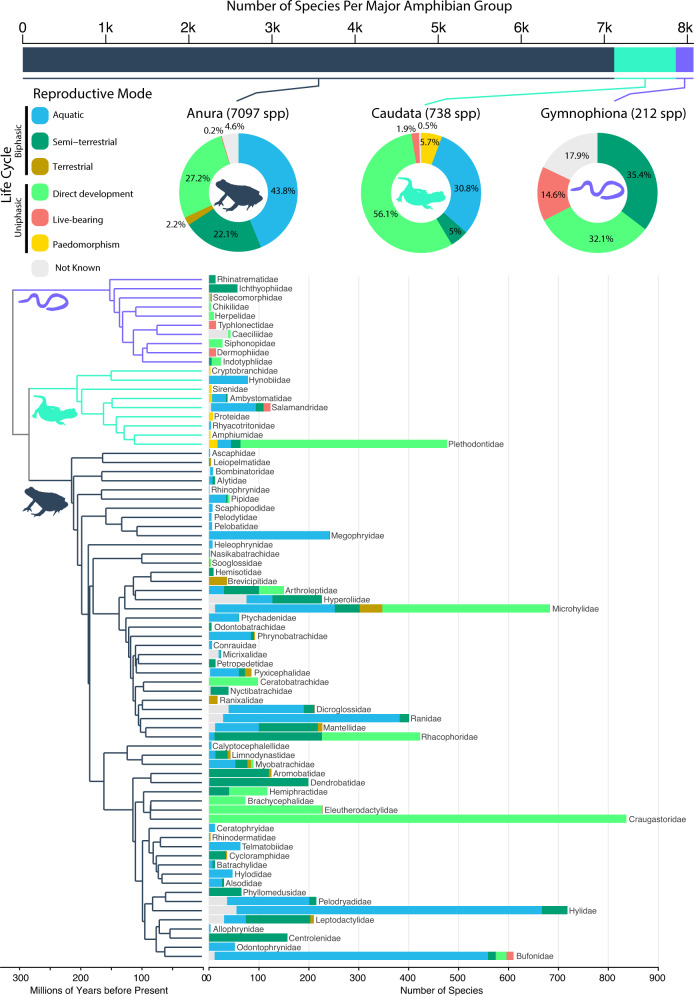
Phylogenetic distribution of reproductive modes in amphibians. Species richness per amphibian group (top bar chart where Anura: dark blue gray, Caudata: mint green, Gymnophiona: lilac, and 1k = 1000 species) and frequencies of reproductive modes per group (percentages as pie charts), and per family (numbers of species as stacked bars on phylogeny). Reproductive modes are represented as blue = aquatic; dark green = semi-terrestrial; brown = terrestrial; light green = direct development; red = live-bearing; yellow = paedomorphism. Frequencies and percentages of unknown reproductive modes are depicted in light gray. The phylogeny shows the relationships of all 75 amphibian families, with icons and branch colors highlighting the three different amphibian clades. Source data are provided as a Source Data file.

Amphibians are an excellent system for comparing life-cycle evolution across major groups. For all three groups, the ancestral reproductive mode is thought to include aquatic larvae^5,6^, which is retained by numerous species in all groups^6^ (Fig. 1). All three groups also have many species lacking the larval stage (direct development) and some species in which females give birth to live young^7^ (viviparity; Fig. 1). However, these three clades also differ in many respects, including body form (tailless frogs, limbless caecilians, and tailed and limbed salamanders), species richness (~7100 frogs, ~700 salamanders, ~200 caecilians), and overall geographic distributions (caecilians are mostly tropical, most salamander families are temperate, and frogs are distributed globally)^8,9^. These differences extend to reproductive modes as well. Bearing live young is rare in frogs and salamanders but more common in caecilians^7^. Paedomorphism, defined very narrowly here as the absence of a distinct postmetamorphic adult stage, is absent in frogs and caecilians but occurs in most salamander families^10^. Life cycles with aquatic eggs have been completely lost in caecilians^4^. Frogs have greater diversity of reproductive modes than the other groups^4^.

**Fig. 2:**
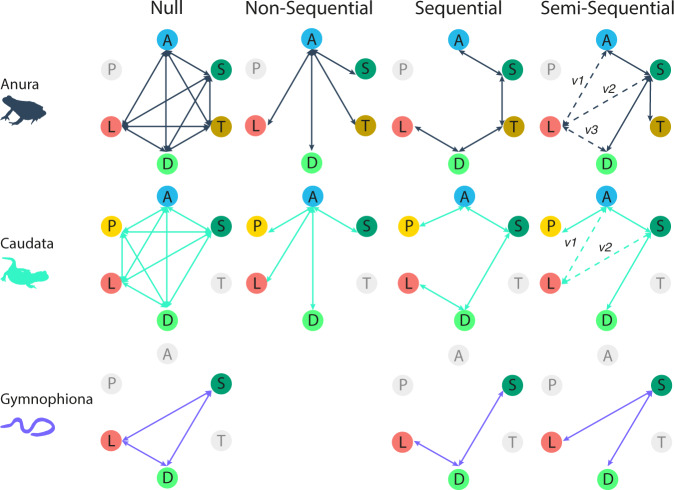
Schematics of four scenarios of reproductive-mode evolution. Null: transitions between all states (reproductive modes) can occur. Non-sequential: derived (more terrestrial) states can only transition to and from the ancestral aquatic mode. Sequential: transitions can only occur between the ecologically most similar states. Semi-sequential: derived, fully terrestrial modes (direct development, live bearing and fully terrestrial biphasic) can only transition to and from the semi-terrestrial mode. Up to three variations of this final scenario were tested (dashed lines), allowing live-bearing to transition either to and from the aquatic mode, the semi-terrestrial mode, or direct development. Node colors and annotations reflect reproductive modes, where A, blue = aquatic; S, dark green = semi-terrestrial; T, brown = terrestrial; D, light green = direct development; L red = live-bearing; P, yellow = paedomorphism. Network edge colors and icons distinguish the three amphibian groups, where Anura: dark blue-gray, Caudata: mint green, Gymnophiona: lilac. Because not all six states are present in all three major clades of amphibians (nodes in gray), the scenarios vary between them.

Much diversity in amphibian reproductive modes involves modifications in their life cycles, such as the presence or absence of whole life stages, and whether embryonic and larval development occur in water or on land^11–13^. These two aspects are often tightly linked. For example, most oviparous species that have lost the larval stage (direct development) lay terrestrial eggs^4^. Amphibian reproductive modes are traditionally thought to have evolved sequentially with only single ontogenetic modules (life stages) changing at each time^4,14,15^. Following this view (Fig. 2), the ancestral mode is biphasic, fully aquatic development^16^. The first step is the evolution of a terrestrial egg, but maintaining an aquatic larval stage, followed by a terrestrial larval stage, before losing the larval stage to become direct developing (i.e., post-metamorphic juveniles hatching from eggs). Live-bearing is the most derived mode, in which embryos are retained (with or without the egg) in the oviduct of the mother until metamorphosis or late stages of larval development.

The sequential hypothesis has been partially tested for several amphibian clades. Transitions to more terrestrial modes appear to have occurred sequentially in some clades (e.g., rhacophorid^17^ and afrobatrachian^18^ frogs, caecilians^19^), but not others (e.g., leptodactylid frogs^20^). Most importantly, a study of 720 frog species (~10% of anurans) suggested that direct development may have evolved directly from fully aquatic ancestors as frequently as from intermediate semi-terrestrial modes^21^. However, this hypothesis has not been tested across all major groups of amphibians. Moreover, few alternatives to strictly sequential versus strictly non-sequential scenarios (where derived traits all evolve from the ancestral state directly) have been explored. For example, evolving a terrestrially adapted egg may be an evolutionary precursor for completely terrestrial life cycles with terrestrial eggs (e.g., direct development), but not for life cycles without eggs (e.g., live-bearing). Scenarios where only modes that have shared features (e.g., terrestrial eggs) have evolved sequentially therefore need to be considered. There may also be reversals from more terrestrial modes to more aquatic modes^21^, which would be inconsistent with the traditional sequential scenario of increasing terrestriality.

We also need to understand how transitions among reproductive modes and life cycles may have impacted diversification. The observation that some of the most species-rich amphibian clades have direct development has led to speculation that this mode may drive increased diversification rates^22–25^. Diversification rate is the rate of species accumulation over time, or the speciation rate minus the extinction rate. The independence of direct-developing lineages from aquatic habitats may have facilitated their expansion into new niche space (e.g., arboreal microhabitats in frogs and salamanders). Occupation of new niche space can lead to ecological opportunity^26^ and thus to increased diversification rates through reduced extinction and/or increased speciation. Furthermore, increased terrestriality is generally accompanied by increased egg size, decreased clutch size, reduced body size^21^, and increased parental care^21,27–29^. These changes, especially reduced body size, may reduce dispersal ability and thereby increase diversification, by increasing allopatric speciation^30,31^. However, reduced dispersal ability might decrease long-term diversification instead^32^, since smaller clade-level range sizes might decrease opportunities for allopatric speciation and make species more susceptible to extinction. The idea that reproductive mode influences diversification has seldom been tested in amphibians at a broad scale. Relevant studies have provided evidence for this hypothesis (e.g., Rhacophoridae^33^) and against it (e.g., *Phrynobatrachus*^34^ and phytotelma-breeding frogs^35^). A study across frogs found that reproductive modes did not significantly impact diversification, but had limited species-level sampling^21^. Two studies in salamanders found that paedomorphosis significantly decreases diversification rates^36,37^, with one study on plethodontids^37^ linking this pattern to reduced geographic range sizes in paedomorphic species (which have limited ability to disperse overland). In summary, there is some equivocal evidence that terrestrial reproduction might increase diversification in frogs and salamanders, and some evidence that changes in life cycle through paedomorphosis decreases diversification in salamanders, but large-scale tests have been limited overall.

There is extensive literature on amphibian reproductive-mode evolution, but core aspects remain untested across the major amphibian groups. Here, we address three open, long-standing questions by taking advantage of a relatively complete, amphibian-wide phylogeny, advanced trait-evolution models, and a growing knowledgebase on amphibian reproductive modes. First, how frequent are different reproductive modes among species within and among major amphibian clades (frogs, salamanders, and caecilians)? Second, do reproductive modes evolve sequentially, from more aquatic to more terrestrial, or in a more complex way? Third, do different reproductive modes or life cycles differentially impact rates of diversification? More broadly, we ask whether these patterns of reproductive-mode evolution are shared or differ among these groups.

## Results

### Distribution of reproductive modes across amphibians

We performed a literature survey and coded reproductive modes for 95% of described, extant amphibian species (7681 of 8047; Supplementary Data 1). These included 95.4% of anuran species, 99.5% of caudates, and 82.1% of gymnophionans. We categorized each species into one of six modes (Fig. 1): aquatic (aquatic eggs and larva), semi-terrestrial (terrestrial eggs, aquatic larva), terrestrial (terrestrial eggs and larva), direct developing (terrestrial eggs, no larva), live-bearing (no eggs, no larva), or paedomorphic (no adult stage).

Surprisingly, the majority of sampled amphibians lay eggs on land, not water (Anura = 51.5% of *n* = 7097 sampled species in total; Caudata = 61.1% of *n* = 738 species; Gymnophiona = 67.5% of *n* = 212 species; Fig. 1). In anurans, biphasic fully aquatic reproduction (eggs and larvae develop in water) was the most common mode, present in 43.8% of sampled species. In contrast, direct development was the most common mode in Caudata (56.1% of species), but was present in only 27.2% of Anura. In Gymnophiona, the most common states were semi-terrestrial biphasic (35.4%; eggs on land, but larvae develop in water) and direct development (32.1%). Semi-terrestrial, biphasic reproduction was also widespread in Anura (20.9%), but not Caudata (5%). Obligate paedomorphism was present only in Caudata, and was relatively rare (5.7%). Live-bearing was rare in Anura (0.2%) and Caudata (1.9%), and more common in Gymnophiona (14.6%).

### Reproductive-mode evolution

We confirmed that the most recent common ancestor of amphibians most likely had aquatic larvae that hatched from aquatic eggs (Supplementary Figs. 7 and 9). We then tested four scenarios for how more terrestrial modes evolved within each group, by constraining transition rates (Fig. 2). These models were: (1) transitions between all modes are possible (null scenario); (2) all derived modes evolve directly and independently from a fully aquatic ancestral state (non-sequential scenario); (3) modes evolve sequentially from most aquatic to least aquatic (sequential scenario); and (4) semi-terrestriality (terrestrial eggs with aquatic larvae) precedes other modes with terrestrial eggs (fully terrestrial and direct development), but transitions are otherwise unordered (semi-sequential scenario). For all models, transitions were allowed to be bidirectional. We compared the fit (using Akaike Information Criterion [AIC]^38^ and Akaike weights [AICw]^39^) among scenarios by fitting hidden Markov models of discrete character evolution with the R package corHMM^40^. We tested different rate restrictions (equal, symmetric or all-rates-different; see Methods) and the inclusion of hidden states to allow for rate variation within observed states. This resulted in up to 36 models per group (full model-fitting results in Supplementary Tables 1–4).

**Fig. 3:**
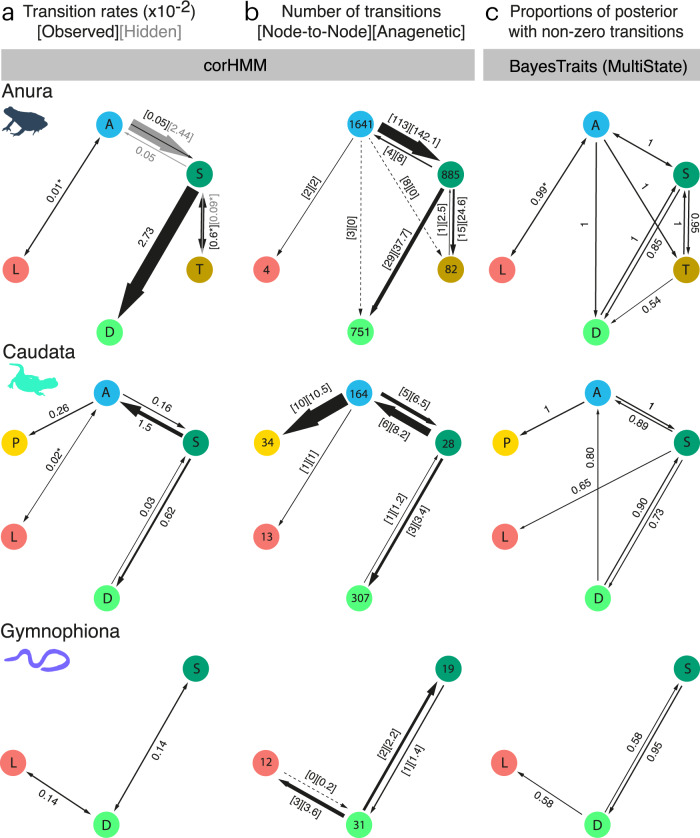
Estimated rates, frequencies and posterior sampling of reproductive-mode transitions in each major amphibian group. **a** Estimated transition rates (×10^−2^) from best-performing corHMM models. Gray arrows and annotations represent hidden state rates. Rates lower than 0.01 are not shown. The best models for Anura and Caudata allowed for all rates to be different (except for pairs of rates annotated with an asterisk that have been forced to be symmetrical; see Methods), and the model for Gymnophiona had all rates set to be equal. **b** Numbers of transitions when estimating ancestral states at nodes only using joint estimations [Node-to-Node] and the mean numbers of transitions along branches [Anagenetic], estimated from 1000 stochastic character maps. Dashed lines indicate transitions only recovered using one of the two methods. The number of species per reproductive mode represented in the phylogeny are indicated in the nodes of the networks. **c** Proportions of models in the posterior where a given transition was estimated (i.e., non-zero transition rate) by the reverse jump MCMC of the MultiState Covarion algorithm implemented in BayesTraits. Proportions of 1 indicate transitions that were present in all models sampled by the MCMC. Transitions with proportions <0.5 are not shown. In all cases, color, position and letters of nodes are consistent and represent A, blue = aquatic; S, dark green = semi-terrestrial; T, brown = terrestrial; D, light green = direct development; L red = live-bearing; P, yellow = paedomorphism. Arrow weight is roughly proportional to transition rates (not to scale). Icons for each panel row distinguish the three amphibian groups. Source data are provided as a Source Data file.

The best-fitting scenario for Anura was semi-sequential (AICw = 0.732; Supplementary Table 1 and Fig. 3). Here, the semi-terrestrial mode evolved from the ancestral aquatic mode, and gave rise to the other two modes with terrestrial eggs: fully terrestrial and direct development (semi-sequential scenario 1 in Fig. 2). Live-bearing evolved directly from aquatic ancestry. This model also allowed for different rates across transitions (all-rates-different: ARD) and within-state transition-rate variation, modeled as hidden states. One other model had AICw>0.01 (AICw = 0.267, ΔAIC = 2.019; Supplementary Table 1). This model differed in that live-bearing evolved from the semi-terrestrial mode, not the aquatic mode (semi-sequential scenario 2 in Fig. 2).

The same scenario (semi-sequential scenario; Fig. 2) also had the best fit in Caudata (AICw = 0.454; Fig. 3). For Caudata, nine models fell within AICw > 0.01 and two with ΔAIC < 2 relative to the best-fit model (Supplementary Table 2). Of these nine models, seven were variants of the same scenario but with different rate restrictions and with live-bearing evolving directly from the aquatic or semi-terrestrial states (semi-sequential scenarios 1 or 2 in Fig. 2).

For Gymnophiona, a purely sequential scenario (sequential scenario in Fig. 2) with no hidden states and equal transition rates was the top-ranking model (AICw = 0.421; Fig. 3). A variant of this scenario with symmetrical rates fell within ΔAIC < 2 (ΔAIC = 1.88, AICw = 0.164; Supplementary Table 3) and 11 models had AICw > 0.01.

To test if we overlooked important alternative models, we also conducted a hypothesis-free, model-reduction analysis using the MultiState Reversible-Jump Markov chain Monte Carlo (rjMCMC) implementation of BayesTraits V4^41^. This method reduces the model complexity of the full transition-rate matrix (“null scenario” in Fig. 2), by restricting some transition rates to be zero or equal to each other. These simpler transition models are then sampled in direct proportion to their fit in the MCMC^42^. Transitions that are not well supported by the data are therefore more frequently set to zero in the posterior. The proportions of times the different transitions were sampled in the posterior (i.e., non-zero) resulted in transition models that was largely congruent with the best-performing corHMM models for each group (Fig. 3c). However, for Anura transitions from fully aquatic to both the fully terrestrial mode and direct development were also supported (present in 100% of the posterior), as were transitions from direct development to the semi-terrestrial mode and from the terrestrial mode to direct development. This last transition was only present in 54% of the posterior, however. For Caudata, the BayesTraits analysis supported transitions from direct development to the aquatic mode and transitions from semi-terrestrial to live-bearing. The latter was represented in 65% of the posterior and therefore arguably ambiguous. Nonetheless, no transition from aquatic to live-bearing was supported. Thus, the transition scenario from BayesTraits was more similar to the second-best corHMM model where this transition was also recovered (Supplementary Table 2; semi-sequential scenario 2 in Fig. 2).

### Rates and frequencies of reproductive-mode transitions

The best-performing model for Gymnophiona had identical transition rates fixed between states. For Anura and Caudata, the best-performing models had variable rates, with the highest transition rates away from the semi-terrestrial mode (Fig. 3a). For Anura and Caudata, rates to and from live-bearing (fixed to be symmetrical due to methodological limitations; see Methods) were very low. The lowest rate in Caudata was from paedomorphism to aquatic and in Anura from direct development to semi-terrestrial (all rates <0.001 events/million years [Myr]).

We used these best-fit models (Supplementary Tables 1–3) to estimate frequencies of reproductive-mode transitions across trees (Fig. 3b). We applied two approaches. We used joint ancestral-state estimates to count only transitions between nodes, and stochastic-character mapping (SCM) to count mean anagenetic transitions. The latter can capture multiple transitions along a single branch (see phylogenies with reconstructed states in Supplementary Figs. 1 and 2). In both cases, frequencies are contingent on the model used to estimate them. For Caudata and Gymnophiona, the two methods result in largely congruent estimates of transition frequencies and patterns, but for Anura important discordances exist (Fig. 3b).

For Anura (Fig. 3b), regardless of the method used, semi-terrestriality evolved most frequently (114 times from joint estimates; 144.6 from SCM), followed by direct development (32 versus 37.7). Live-bearing evolved least frequently (2 times). The joint estimates suggested that the complete terrestriality and direct development sometimes evolved directly from the ancestral, aquatic biphasic mode (8 and 3 times, respectively), but these transitions were not found using SCM.

In Caudata (Fig. 3b), paedomorphism evolved most frequently (10 times, both methods), and always from the aquatic mode. Semi-terrestriality evolved many times (joint estimation = 6; SCM = 7.7), mostly from an aquatic ancestor, but with one reversal from direct development. The terrestrial egg was lost frequently through reversals to the fully aquatic mode (joint estimation = 6; SCM = 8.2). Direct development evolved three times, always from a semi-terrestrial ancestor, with one estimated reversal (loss). Live-bearing evolved only once, from an aquatic ancestor.

In Gymnophiona (Fig. 3b), the most frequent transitions were from direct development to live-bearing (joint estimation = 3; SCM = 3.64). Reversals from direct development to semi-terrestrial were twice as common as origins of direct development. There were no reversals from live-bearing.

### State-dependent diversification

We used extensions of the State-dependent Speciation and Extinction (SSE) framework^43,44^ to test whether different reproductive modes or life cycles were associated with different diversification rates (speciation minus extinction). We used the hisse^45^ and secsse^46^ R packages, which both allow for multi-state and hidden-state modeling (but each with limitations, see Methods). We used hisse to test whether rate shifts occurred in association with shifts in life cycle, specifically the loss of the larval phase (direct development and live-bearing) or adult phase (paedomorphism). We used secsse to test rate shifts associated with reproductive modes (five states for Anura and Caudata, three for Gymnophiona). For both analyses we tested the fit (based on AIC and AICw) of three scenarios: (1) diversification rates are constant across the phylogeny; (2) diversification rates vary with reproductive mode (with or without rate variation across hidden states); and (3) diversification rates vary, but only across unmeasured (hidden) trait states, not across the observed reproductive modes. The parameters for the best-fit models for all three groups are provided as Supplementary Data 2.

Diversification rates varied across the phylogeny in Anura and Caudata, but not Gymnophiona. This rate variation was attributable to shifts in reproductive modes in Caudata, but not Anura. In anurans, variable rates were due to other, unmeasured (i.e., hidden) traits (Tables 1 and 2). This was the case regardless of the method and trait classification used (hisse with up to three life cycles, and secsse with up to five reproductive modes).

**Table 1:** Ranking of model performance of state-dependent speciation and extinction “hisse” models for the three major groups of amphibians

Diversification rate model	Rate categories	lnL	AIC	ΔAIC	AICw
**Anura**					
Reproductive-mode Independent	4	−13,955.550	27,933.100	0.000	1.000
Reproductive-mode Dependent	4	−13,949.151	27,948.302	15.202	0.000
Reproductive-mode Independent	3	−13,977.643	27,973.287	40.187	0.000
Reproductive-mode Dependent	3	−13,970.869	27,979.738	46.638	0.000
Reproductive-mode Dependent	2	−14,021.170	28,068.340	135.240	0.000
Reproductive-mode Independent	2	−14,028.462	28,070.924	137.824	0.000
Reproductive-mode Dependent	1	−14,227.051	28,466.101	533.001	0.000
Constant	1	−14,235.512	28,479.025	545.925	0.000
**Caudata**					
Reproductive-mode Dependent	2	−2054.922	4159.844	0.000	0.990
Reproductive-mode Dependent	3	−2047.560	4169.121	9.277	0.010
Reproductive-mode Independent	4	−2075.380	4180.760	20.916	0.000
Reproductive-mode Independent	3	−2077.676	4181.352	21.509	0.000
Reproductive-mode Dependent	4	−2044.562	4187.124	27.280	0.000
Reproductive-mode Independent	2	−2089.255	4200.510	40.666	0.000
Reproductive-mode Dependent	1	−2095.557	4215.114	55.270	0.000
Constant	1	−2143.645	4303.290	143.446	0.000
**Gymnophiona**					
Constant	1	−319.401	646.803	0.000	0.809
Reproductive-mode Dependent	1	−319.327	650.654	3.851	0.118
Reproductive-mode Independent	2	−319.002	652.004	5.202	0.060
Reproductive-mode Independent	3	−318.934	655.869	9.066	0.009
Reproductive-mode Dependent	2	−315.992	657.984	11.181	0.003
Reproductive-mode Independent	4	−318.921	659.843	13.040	0.001
Reproductive-mode Dependent	3	−315.436	668.872	22.069	0.000
Reproductive-mode Dependent	4	−315.190	680.381	33.578	0.000

Models fall into three categories: Constant diversification (CD), Reproductive-mode Dependent Diversification (RmDD; state-associated rate shifts, with or without hidden traits), Reproductive mode Independent Diversification (RmID; rate shifts only associated with states of hidden traits, not observed reproductive modes). RmDD models can have different numbers of hidden traits, resulting in different numbers of rate categories.

*lnL* log likelihood, *AIC* Akaike information criterion, *ΔAIC* difference between AIC of each model and the best-fit model, *AICw* Akaike weights.

**Fig. 4:**
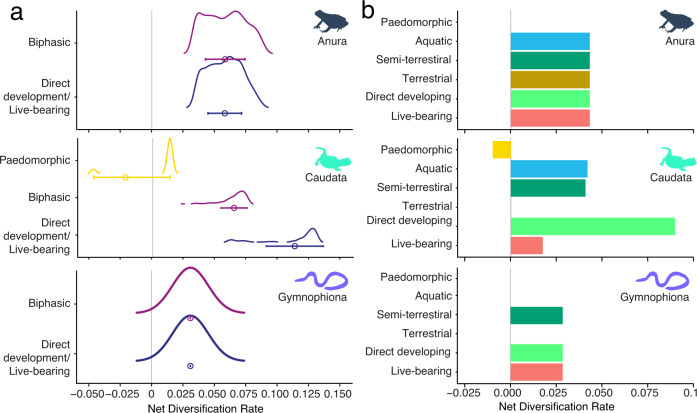
Net diversification rates (speciation minus extinction) per reproductive modes for Anura, Caudata and Gymnophiona. **a** Distributions (with standard deviation error bars around the mean) of rates estimated per species in the phylogeny for each life-cycle category (i.e., tip rates). Species are categorized into biphasic life cycles (dark magenta), direct development (dark blue) or paedomorphism (yellow). Sample sizes (where *n* = number of species) for each are Anura: biphasic = 2608, direct development = 755; Caudata: biphasic = 192, direct development = 320, paedomorphism = 34; Gymnophiona: biphasic = 19, direct development = 43. Estimates were calculated using the best-fitting hisse models, which for Anura was a Reproductive mode Independent Diversification rate model (RmID) with hidden states, for Caudata was a Reproductive mode dependent Diversification rate model (RmDD) with hidden states, and for Gymnophiona was a Constant Diversification rate model (CD). In cases where models contained hidden states, rates are weighted averages per species. **b** Diversification rate estimates for each of the six reproductive modes, where blue = aquatic; dark green = semi-terrestrial; brown = terrestrial; light green = direct development; red = live-bearing; yellow = paedomorphism. Estimates are calculated using the best-fitting secsse models, which for Anura was a Reproductive mode Independent Diversification rate model (RmID) with hidden states, for Caudata was a Reproductive mode dependent Diversification model (RmDD) with hidden states, and for Gymnophiona was a Constant Diversification rate model (CD). In cases where models contained hidden states, rates shown are averaged across hidden and observed states. Icons in both panels distinguish the three amphibian groups. Source data are provided as a Source Data file.

For anurans, the state-independent (variable) models outperformed all others (hisse: AICw = 1.000; secsse: AICw = 0.852; Tables 1 and 2). The secsse analysis estimated a diversification rate of 0.080 species/Myr across all reproductive modes when averaging across hidden and observed state rates (Fig. 4b and Supporting Data 2). Estimating tip (i.e., extant species) net diversification rates using the hisse model yielded similar rate distributions for biphasic and direct-developing species, centered on mean rates of 0.058 species/Myrs for both (sd = 0.016 and sd = 0.013 respectively; Fig. 4b).

**Table 2:** Ranking of model performance of state-dependent speciation and extinction “secsse” models for the three major groups of amphibians

Diversification rate model	Rate categories	lnL	AIC	ΔAIC	AICw
**Anura**					
Reproductive-mode Independent	2	−14,598.183	29,218.365	0.000	0.852
Reproductive-mode Dependent	2	−14,577.932	29,221.865	3.500	0.148
Constant	1	−14,802.135	29,620.269	401.904	0.000
Reproductive-mode Dependent	1	−14,796.597	29,625.193	406.828	0.000
**Caudata**					
Reproductive-mode Dependent	2	−2092.489	4254.979	0.000	1.000
Reproductive-mode Independent	2	−2124.984	4273.968	18.989	0.000
Reproductive-mode Dependent	1	−2134.717	4303.434	48.455	0.000
Constant	1	−2180.131	4378.262	123.283	0.000
**Gymnophiona**					
Constant	1	−333.016	678.033	0.000	0.900
Reproductive-mode Dependent	1	−331.907	683.815	5.782	0.050
Reproductive-mode Independent	2	−332.909	683.818	5.785	0.050
Reproductive-mode Dependent	2	−328.233	698.466	20.433	0.000

Models fall into three categories: Constant diversification (CD), Reproductive-mode Dependent Diversification (RmDD; state-associated rate shifts, with or without hidden traits), Reproductive mode Independent Diversification (RmID; rate shifts only associated with states of hidden traits, not observed reproductive modes). RmDD models can have different numbers of hidden traits, resulting in different numbers of rate categories.

*lnL* log likelihood, *AIC* Akaike information criterion, *ΔAIC* difference between AIC of each model and the best-fit model, *AICw* Akaike weights.

In Caudata, the best-performing models were reproductive-mode dependent models with hidden states, for both hisse and secsse (AICw = 0.990 and AICw = 1.000 respectively; Tables 1 and 2). When estimating tip net-diversification rates with this best-performing hisse model, direct-developing caudates had the highest mean net-diversification rates (mean = 0.113 species/Myr, sd = 0.023; Fig. 4a). Biphasic species had intermediate rates (mean = 0.065, sd = 0.034), and paedomorphic species had the lowest rates (mean = −0.022, sd = 0.010). Rate distributions were generally multi-modal or skewed (due to variation in rates across hidden-trait states), and therefore mean rates should be interpreted with caution. The secsse model estimated the highest rates (averaged across observed and hidden states) for direct development (0.094 lineages/Myr) followed by aquatic (0.044), semi-terrestrial (0.043), live-bearing (0.019), and paedomorphism (−0.010; Fig. 4b and Supporting Data 2).

The best-performing models for Gymnophiona were constant-rate models for both hisse and secsse (hisse: AICw = 0.809; AICw = secsse: 0.900; Tables 1 and 2). All species were therefore estimated to diversify at 0.031 species/Myr with hisse, and 0.029 with secsse.

## Discussion

Here we provide a large-scale phylogenetic comparison of macroevolutionary patterns in life-cycle and reproductive-mode evolution among the three major groups of amphibians. We specifically focused on comparing species richness of different reproductive modes, testing alternative evolutionary scenarios of transitions among modes, and variation in diversification rates associated with each mode.

### Frequencies and transitions of reproductive modes

Our compilation of reproductive modes, the largest for amphibians to date, shows that the three groups have different patterns of species richness among reproductive modes and life cycles (Fig. 1). Frogs and salamanders have many species with fully aquatic reproductive modes, whereas caecilians have none. Salamanders have paedomorphic species, whereas the other two groups do not. Salamanders are dominated by species with direct development and have very few semi-terrestrial species: both frogs and caecilians have similar proportions of species with direct development and with semi-terrestrial reproduction. Caecilians have many live-bearing species, whereas salamanders and frogs have very few.

At the same time, there are important similarities among groups in these richness patterns. For example, all three groups have many species with direct development. All three groups have many species retaining the most ancestral mode. No groups are dominated by live-bearing (Anura: 0.2%, Caudata: 1.9%, Gymnophiona: 14.6%) or paedomorphic species (5.7% in Caudata, 0% in all others). This is particularly interesting given that in caudates the most frequent transitions are gains of paedomorphism, and in gymnophionans gains of live-bearing.

Our ancestral-state reconstructions found that the most recent common ancestors for all three amphibian groups had biphasic life cycles that were subsequently lost multiple times. Alternative larval stages such as non-feeding lecithotrophy, have evolved multiple times across metazoans^47,48^, and evolutionary losses of larval stages have occurred frequently^49,50^. The re-evolution of lost, complex traits (such as entire life-stages) was long thought to be impossible. However, a growing number of studies suggest otherwise^51,52^. The re-appearance of the larval stage after its loss may have occurred in frogs^53^, salamanders^54^ and caecilians^19^. In line with these studies, we found support for reversals from direct development in salamanders and caecilians, with some support in frogs (in the BayesTraits analysis). The detection of reversals can be influenced by the models and methods used for ancestral-state estimations and by the taxon sampling and phylogenetic trees used^53,55^. For example, previous studies found re-appearance of the larval stage in hemiphractid frogs^53,55^, which we did not recover with our ancestral-state reconstructions based on corHMM models. This is possibly because rate heterogeneity within hemiphractids may make this pattern difficult to detect^53^, a problem that would be exacerbated when analyzing all frogs simultaneously. The re-appearance of the larval stage in Anura, Caudata, and Gymnophiona is therefore possible, but rare. In all three groups, transition frequencies are exceedingly asymmetrical, favouring loss of the larval stage. The ontogeny of different direct-developing lineages^56,57^ may explain why so few direct-developing clades have had reversals. For example, direct-developing hemiphractid frogs have retained some biphasic embryonic features (e.g., oral structures) inside the egg^53,58^. The same is true for the reversal in *Desmognathus* salamanders^59^ and Seychellean caecilians^60^. Reversals may therefore be viable only if key larval features were retained. Moreover, there is discussion over whether these apparent reversals represent true “re-evolution” of larval stages or further elaborations of existing developmental sequences^59^. Studies on the regulatory genomic changes required to evolve direct development or live-bearing may therefore be crucial for understanding how these transitions occur and why they are seldom reversed^61,62^.

### Reproductive-mode evolution

We used both hypothesis-driven (corHMM) and hypothesis-free (BayesTraits) approaches to investigate reproductive-mode evolution in amphibians. Caecilians were the only group in which evolution proceeded sequentially from most aquatic to least aquatic (semi-terrestrial to direct developing to live-bearing), as predicted^4^. In Anura and Caudata, the sequential models were not among the best-fitting scenarios (AICw ≤ 0.01; Supplementary Tables 1 and 2). Lineages with terrestrial larvae did not give rise to direct-developing lineages (transition absent in the best-fit corHMM model and only weakly supported by BayesTraits) and direct developers did not evolve live-bearing. Instead, these three modes represent end-points of different trajectories for evolving terrestrial life cycles. Congruently, although all three modes are ecologically similar (minimal dependency on aquatic habitats), they have diverged extensively in their developmental biology^15,63^.

A crucial sequential element is maintained in all three groups: the semi-terrestrial mode appears to have been a frequent precursor for direct-development. Our best-fit, semi-sequential hypothesis is therefore congruent with previous reports of sequential evolution in the frog clades Rhacophoridae^17,33^ and Afrobatrachia^18^. Neither of these clades have live-bearing species and they show only that terrestrial eggs evolved prior to terrestrial (or late hatching) larvae. Our hypothesis is also congruent with the reported non-sequential evolution in Leptodactylidae^20^. This study used a more fine-grained categorization of reproductive modes (e.g., distinguishing between foam and non-foam terrestrial nests), in species that fall within only two of our reproductive categories: semi-terrestrial and terrestrial. As such, our more generalized, semi-sequential model also adequately describes reproductive-mode transitions in this group.

Some features associated with the transition to laying eggs on land may therefore have been key in later enabling the evolution of terrestrial larvae and/or direct development. These features may include increased egg size and provisioning^11,21^, desiccation avoidance adaptations^64^, enhanced embryonic gas-exchange capabil﻿ities^65^, and parental care^27,28^. However, some of these characteristics may not be a requirement for live-bearing, in which neither the egg nor the developing embryo are exposed to the environment. Hence, in frogs, live-bearing seems to have evolved separately. The evolution of live-bearing in salamanders remains equivocal, evolving from fully aquatic or semi-terrestrial ancestry. In caecilians, the origin of live-bearing from direct development is strongly supported across methods.

Discordances in the evolution of live-bearing across clades merits further investigation. Overall, the evolution of live-bearing in vertebrates seems unlikely to conform to a single scenario^66^. For example, some live-bearing salamanders^67^ and frogs^68^ give birth to individuals in advanced larval stages. The modeling of live-bearing evolution may also be influenced by uncertainties in reproductive-mode coding and limited phylogenetic sampling of key species. For instance, there is some uncertainty around the reproductive modes of the cave salamander (*Speleomantes sarrabusensis*) that may be capable of bearing live young^69^ (but see ref. 70). There is also uncertainty about the reproductive mode of the closest living relatives of the live-bearing anuran genera *Nectophrynoides* and *Nimbaphrynoides*^71^. Furthermore, a possible third origin of live-bearing in frogs has been reported in the now extinct *Eleutherodactylus jasperi*, in a genus that is otherwise direct developing^72^.

Previous studies in frogs have found that direct development and terrestrial biphasic development may have evolved directly from fully aquatic ancestors^20,21^. The hypothesis-free BayesTraits analysis supports such direct transitions. Interestingly, we show that such a signal can also be obtained even if such transitions do not occur in the underlying state-transition model. When reconstructing ancestral states based on the best-fitting, semi-sequential corHMM model and estimating transition frequencies between nodes only (using a joint-estimation method), we recovered at least some direct transitions from fully aquatic development to direct development and terrestrial biphasic. However, these transitions did not occur when counting anagenetic transitions (through stochastic character mapping). We cannot rule out the occurrence of direct transitions, but observations of direct transitions may still involve intermediate, semi-terrestrial ancestors, as long as these occur rapidly and along a single branch^21^.

In support of this idea, we find that the transition rates and frequencies estimated for the loss of the larval stage (from semi-terrestrial to direct development) are among the highest of any state transitions in Anura and Caudata. The loss of the larval stage may thus have evolved rapidly through simple regulatory changes that are not easily captured by macroevolutionary methods. It is often a challenge for comparative analyses to reconstruct evolutionary patterns in developmental modes when evolutionary changes are rapid^50^. To understand how such seemingly complex changes in life-cycles can occur rapidly, we may need to gain a better understanding of their genomic and developmental causes. Developmental studies have found that direct development may evolve through heterochronic shifts in key developmental mechanisms, such as the shift in peak thyroid hormone levels during the embryonic period^56,61,73–75^. Similarly, viviparity in *Salamandra salamandra* may have evolved via accelerated development and heterochrony of the feeding and digestive systems^67^.

### Reproductive modes and diversification rates

Our results challenge the idea that direct development or terrestrialization in anurans increase diversification rates^22–25^. To our knowledge, substantial support for this hypothesis has only been found for direct development in a single frog family (Rhacophoridae^33^). Across Anura, we find little evidence to support this claim. We do find heterogeneity in diversification rates within Anura that is attributable to unmeasured (hidden) trait states. Our results do not address what these hidden states are, but previous studies have shown that climate^76–79^, microhabitat^79^, and climatic-niche evolution^78^ impact anuran diversification.

In contrast, we found that salamander species with direct development have higher diversification rates. Direct development is also the most common reproductive mode among salamanders, unlike in frogs and caecilians. Interestingly, direct development has only evolved in a single family, Plethodontidae. This family includes the only salamanders that colonized the Neotropics, where they diversified extensively^80,81^. All tropical plethodontids have direct development, but there are also many temperate plethodontid lineages with direct development (e.g., *Batrachoseps, Plethodon*). The method we used ideally captures rate variation produced by unmeasured, hidden trait states (e.g., biogeography). However, the few origins of direct development in salamanders may make its direct effects on diversification difficult to disentangle from other clade-specific factors.

Intriguingly, we found that paedomorphism was associated with negative diversification rates (extinction rates higher than speciation rates). This is consistent with the numerous repeated origins of paedomorphosis (the most common reproductive-mode transition in Caudata), but the low number of extant paedomorphic species. There is some support for a negative relationship between diversification rates and paedomorphosis among salamander families^36^. Similarly, an analysis of spelerpine plethodontids^37^ found that paedomorphosis increased speciation rates but increased extinction rates even more, leading to decreased net diversification rates. We also find this same speciation-extinction dynamic resulting in negative diversification rates here (Supplementary Data 2). We therefore strongly support the hypothesis that paedomorphosis can decrease diversification.

Unlike Anura and Caudata, we estimated diversification rates to be low and constant across Gymnophiona. Diversification rates in Gymnophiona may also be largely constant over time^82^. Why this may be remains unclear. There is a trend toward lower diversification rates in fossorial frogs^79^ (but not significant) and fossorial squamates^83,84^. Fossoriality is common in caecilians^4^, and might explain their relatively low and constant diversification rates.

In conclusion, here we present a large-scale comparison of evolution of life cycles and reproductive modes among the three major amphibian clades. We found that transition rates among states are largely decoupled from their species richness. Specifically, we found that in all three groups, the most frequent transitions were to states that are relatively uncommon in that group (live-bearing in caecilians, paedomorphosis in salamanders, semi-terrestriality in frogs). All three groups showed numerous transitions to various derived (usually more terrestrial) reproductive modes, but all three contained substantial numbers of species (33–44%) that retained the most ancestral mode. All three groups also showed many reversals to more ancestral modes (i.e., more aquatic). We found that a semi-sequential pattern of reproductive-mode evolution was supported in all three groups, with the evolution of the terrestrial egg most likely preceding the evolution of direct development. We also found that diversification rates are largely decoupled from reproductive modes in amphibians. The major exception was in salamanders, in which direct development appeared to accelerate diversification (which explains why most salamanders have direct development) and paedomorphosis decreased diversification rates (which explains why paedomorphosis remains rare in salamanders, despite numerous origins of paedomorphosis). Many patterns found here might apply to other organisms with complex life cycles, including the decoupling of transition rates from patterns of species richness among reproductive modes (i.e., many transitions to relatively uncommon modes), the widespread retention of the most ancestral modes, and the overall independence of reproductive modes and diversification.

We suggest that one of the biggest challenges for similar studies in amphibians is to understand the processes underlying these patterns of reproductive-mode evolution^85–88^. The most general pattern is the frequent origins of non-aquatic reproduction, including the many species with direct development in all three groups. We know little about the developmental and genomic changes underlying the diversity of reproductive modes.

## Methods

This study contains no experimental component or data collection on live animals and so no ethical oversight was necessary.

### Data collection

We used the time-calibrated molecular phylogeny of Jetz and Pyron^82^ for our phylogenetic analyses. They estimated a phylogeny for 4061 amphibian species based on DNA sequence data. The taxonomy used in that study was adapted to follow the Amphibian Species of the World (ASW) online reference^9^ using the AmphiNom package^89^ in R v3.6.1^90^. Changes were made to either update species names or prune them from the phylogeny if they represent junior synonyms or otherwise invalid names according to this reference. Moreover, two species, *Ceuthomantis smaragdinus* and *Allobates ranoides*, were removed as their inclusion results in the polyphyly of families. In total, 496 species names were changed and 36 species were pruned completely (see Supplementary Data 3). The final tree (Supplementary Data 4) contained 4025 of the 8047 species listed on ASW on the 21st of August 2019 (48.1% of Anura, *n* = 3416 species, 74.0% of Caudata, *n* = 546; 29.7% of Gymnophiona, *n* = 63). The tree included representatives of all 75 amphibian families.

Reproductive modes were scored for as many species as possible using online resources including AmphibiaWeb^8^, IUCN Red List^91^, AmphibiaChina^92^, and Anfibios del Ecuador^93^. If these sources lacked data for a given species, other primary and secondary literature were used (references in Supplementary Data 1). The reproductive mode of each species was coded as being either: aquatic, semi-terrestrial, terrestrial, direct developing, live-bearing, or paedomorphic. Aquatic refers to any biphasic species in which both eggs and larvae develop entirely in water (permanent or ephemeral of any size, including water-filled treeholes). Semi-terrestrial refers to any biphasic species in which eggs are deposited out of water (on land, vegetation, in foam nests, etc.) or in specialized anatomical structures of the adults (pouches, vocal sacs, etc.), but larval development occurs at least partly in water, including species with semi-terrestrial tadpoles. Terrestrial refers to biphasic species in which eggs are deposited out of water, either in nests (foam or otherwise) or in specialized anatomical structures, with larval development also completed out of water. For many of these terrestrial-mode species, larvae are endotrophic and hatch at very late stages in development. Direct development refers to uniphasic development with the absence of the free-living larval phase. Live-bearing refers to development of embryos inside the parent, through the retention or absence of eggs. This broadly encompasses different definitions or types of viviparity such as ovoviviparity, lecithotrophy, matrotrophy, and larviparity^94^. Finally, paedomorphic refers here to species that obligately lack a post-metamorphic adult phase. Supplementary Table 7 describes how these categories relate to those used by Duellman and Trueb^4^.

We recognize that in exceptional cases, species do not fit neatly into our categorization scheme. Firstly, species within a given reproductive mode show variation in their ontogenetic trajectories. For example, there is a broad continuum among adults of paedomorphic salamander species, from those that are largely indistinguishable from larvae of other species (e.g., in ambystomatids and dicamptodontids) to those having many characteristics of metamorphic adults (e.g., cryptobranchids)^4^. The offspring of *Desmognathus aeneus*, considered a direct developing salamander, hatch with mostly post-metamorphic features, but retain external gills, a typical larval feature^95^. In contrast, nidicolous breviceptid frogs have no free-swimming larvae, but are classified as biphasic, terrestrial because metamorphosis occurs in nests, post-hatching^96^. There are also rare cases of species giving birth to larvae (e.g., *Salamandra salamandra*^97^ and *Limnonectes larvaepartus*^68^), which we include here in our broad definition of live-bearing. Secondly, the names of our categories imply that species without aquatic eggs must be partly or fully terrestrial. However, the New World pipid frogs are a clear exception. In these species, eggs are embedded in specialized dorsal tissue of adults (i.e., not strictly aquatic oviposition) from which either fully formed young (e.g., *Pipa pipa*) or free-living larvae (e.g., *Pipa parva*) emerge. With our coding scheme, these are either classified as direct developing or semi-terrestrial, though we realize that all species in this group are ecologically completely aquatic.

In some cases, expert accounts in the online sources were themselves based on indirect lines of evidence such as ovum characteristics or conservatism in modes in all congeners (e.g., direct development within Craugastoridae, or foam-nest building in Rhacophoridae). We adopted such inferences when no contrary evidence was found (*n* = 1254, 15.6% of all species; *n* = 657, 16.3% of all species in the phylogeny). We acknowledge that coding for some of these species may change in the future. In cases where no such inference could be made or expert inferences were questionable, reproductive modes were coded as “NA” to allow for uncertainty to be incorporated in the analyses wherever methods permitted (*n* = 366, 4.5% of all species; *n* = 54, 1.3% of all species in the phylogeny).

All coding is tabulated in Supplementary Data 1. Sample sizes per group per reproductive mode were as follows (species sampled in phylogeny/species sampled for reproductive-mode data): Anura: aquatic = 1641/3107, semi-terrestrial = 885/1565, terrestrial = 82/157, direct development = 751/1928, live-bearing = 4/16, unknown = 53/324; Caudata: aquatic = 164/227, semi-terrestrial = 28/37, direct development = 307/414, Live-bearing = 13/14, paedomorphism = 34/42, unknown = 0/4; Gymnophiona: semi-terrestrial = 19/75, direct development = 31/68, live-bearing = 12/31, unknown = 1/38.

### Reproductive-mode evolution

We first tested the hypothesis of a sequential evolution of reproductive modes, transitioning from more aquatic into more terrestrial. We compared four alternative scenarios of how reproductive modes may have evolved (Fig. 2). (1) A null hypothesis, with no prior predictions, where transitions between all states can occur. (2) A non-sequential, radial hypothesis, where derived states are only allowed to transition directly from the fully aquatic, non-paedomorphic state. (3) A sequential hypothesis, where states can only transition between the next most similar mode, from most aquatic to least aquatic, reflecting the classic idea of sequential or ordered evolution of reproductive modes in amphibians^4^. (4) A semi-sequential hypothesis, in which semi-terrestrial modes are a necessary intermediate step between the aquatic ancestral mode and fully terrestrial modes with eggs (biphasic or direct development). Three variations on this fourth scenario were tested, each representing a distinct scenario for transitions to live-bearing. These included scenarios where live-bearing evolved directly from an aquatic ancestor (version 1), from a semi-terrestrial ancestor (version 2), or from a direct-developing ancestor (version 3). For all hypotheses, paedomorphism always represented an opposite trajectory relative to terrestrialization, arising only from the ancestral, biphasic aquatic mode.

Not all six reproductive modes were represented in each clade and therefore models varied slightly for each. Paedomorphism is only known from Caudata. Biphasic, fully terrestrial life cycles are not known for Caudata or Gymnophiona. For Gymnophiona, no extant species have the fully aquatic mode. In total, this resulted in six hypotheses for Anura, five for Caudata, and three for Gymnophiona (Fig. 2).

We used the package corHMM v2.3^40^ in R to fit and compare these different hypotheses for each amphibian clade separately. Models were constructed by placing restrictions on the transition matrix to prevent transitions not allowed in a given hypothesis. For each hypothesis, three models were fit. Transition rates between pairs of states were either all estimated independently (all-rates-different models, ARD), constrained to be the same (equal-rate models, ER), or constrained so that both transitions between each pair of states had the same rate (rate for transitions from state 0 to state 1 equals the 1-to-0 rate), but that different pairs of states had a different rate (0 and 1 transitions differ from 0 and 2 transitions; symmetric-rate models, SYM).

We performed exploratory analyses in which transitions between states were only permitted in a single direction (i.e., not allowing trait reversals). However, this resulted in corHMM not finding suitable starting parameters for many of these models. Therefore, only models permitting reversals were tested. Preliminary analyses also resulted in problematic results with unreasonably high rates away from states that were only represented by a small fraction of species^98^. This affected transitions away from live-bearing and terrestrial modes in Anura and live-bearing in Caudata (represented by fewer than 5% of species). To offset this problem, transitions to and away from these states were fixed to be the same (i.e., symmetrical).

We re-ran all models after collapsing the terrestrial and semi-terrestrial modes into a single category for Anura to test whether our results were sensitive to this distinction in coding. Re-classification of reproductive modes resulted in the same best-performing model (Supplementary Table 4) and is therefore not discussed further. Likewise, to test the sensitivity of the model fitting to our inclusion of species with unknown or inferred reproductive modes (17.6% of all species in the phylogeny), we repeated the analyses with these species removed. This again had no meaningful effect on the ranking of model performance (Supplementary Table 5).

Multistate character evolution in corHMM can be fitted using a continuous-time Markov model (Mk) and a hidden Markov model (HMM). The latter uses “hidden” states to allow for rate variation within observed states. We fitted both Mk and HMM versions for all models. Overall, we analyzed up to six hypotheses, with three transition-rate models each, and both with and without hidden states (i.e., one or two rate categories). Therefore, we compared a total of 36 models for Anura, 30 for Caudata, and 18 for Gymnophiona (Supplementary Tables 1–3). We ranked the performance of these models using the Akaike Information Criterion^38^ (AIC) and Akaike weights^39^ (AICw).

To confirm that non-aquatic and paedomorphic modes represent derived states in amphibians, we reconstructed ancestral states across the whole amphibian tree using ER, SYM, and ARD transition-rate models, with and without hidden states. Regardless of the choice of root prior (Supplementary Note 1 and Supplementary Table 8), all models estimated an aquatic ancestor (aquatic eggs and larvae) for the most recent common ancestor (crown group) of all extant amphibians, and for the crown-group ancestors of Anura and Caudata (Supplementary Fig. 7). A semi-terrestrial mode (terrestrial eggs, aquatic larvae) was reconstructed for the most recent common ancestor of Gymnophiona (Supplementary Fig. 7). All state-transition scenarios (except the null scenario) require the root states to be either aquatic (for Anura and Caudata) or semi-terrestrial (Gymnophiona). Root probabilities were fixed to these states for all the clade-specific model testing. To reduce the number of parameters estimated, a single rate parameter was set for all transitions between observed and hidden states for the HMM models. We set 25 starting tries to reduce the probability of suboptimal starting parameters. For species in the tree in which states were unknown (*n* = 53 for Anura, *n* = 0 for Caudata, and *n* = 1 for Gymnophiona), equal probabilities across all possible states were assigned to incorporate this uncertainty.

Ancestral-state reconstructions based on the best-performing corHMM model parameters were then used to estimate the frequencies of transitions among reproductive modes. We applied two approaches. (1) We estimated ancestral states for each node in the phylogeny using a joint estimation method^99^ and then summarized the number of times a specific transition occurred between two adjacent nodes (“node-to-node” transitions). (2) We estimated ancestral states using stochastic character mapping^100^ (with 1,000 simulations) and then averaged the number of each transition (“mean anagenetic” transitions). Both approaches were implemented using the corHMM package.

We also explored patterns of evolutionary transitions in reproductive modes using a hypothesis-free, model-reduction approach implemented in BayesTraits V4^41^. BayesTraits can reduce the complexity of transition-rate matrices of a multistate character using Reversible-Jump Markov Chain Monte Carlo (rjMCMC). This is achieved by iterating through models with certain transition rates either restricted to zero or equal to each other. The different models are then sampled in direct proportion to their fit to the data by the MCMC^42^. Exploring which is the most frequently sampled model, as well as the proportions each transition is not set to zero in the posterior, allows identification of the most likely scenarios of trait evolution.

MCMC chains were run three times independently for up to 100 million iterations, sampling an exponential prior with means seeded from a uniform hyperprior ranging from 0 to 100. Chains were sampled every 5000th iteration after an initial burn-in of up to 10,000 iterations. Trees were scaled to have mean branch lengths of 0.001 (to allow better exploration of parameter space^41^). The R package coda v0.19-4^101^ was used to confirm that the chains had reached stationarity with low autocorrelation, converged across runs and showed adequate mixing with effective sample sizes >1000. Analyses were performed independently for Anura, Caudata and Gymnophiona. Transitions to and from live-bearing in Anura had to be restricted to be symmetrical to avoid unrealistic rate estimates due to small sample sizes (see above). No other restrictions were implemented.

We used a covarion model, a variant of the continuous-time Markov model that allows for traits to vary their rate of evolution within and between branches^102^. Using log Bayes Factors (BF), we checked that this resulted in an improvement in model performance over the standard MultiState model. Log BF were calculated using log Marginal Likelihood (log ML) estimates from the stepping-stone sampler in BayesTraits^103^. We set 1000 stones with 5000 iterations each with default parameters. Using log BF = 2(log ML covarion model − log ML simple model), we found log BF = 1.123, 17.801, and 3.933 for Anura, Caudata, and Gymnophiona, respectively (Supplementary Table 9). For all three groups, the covarion model therefore showed an improvement in marginal likelihoods (a positive log BF) over the simpler, rate-homogeneous model, though this improvement was only marginal for Anura (log BF < 2)^41^. The results presented therefore refer to the covarion models. Supplementary Fig. 8 shows the ranking of the most frequently sampled models, for Anura and Gymnophiona, the top model making up 13.9% and 29.0% of the posterior respectively. For Caudata, no single model dominates the posterior, with the highest-ranking model only making up 1.00% of the posterior. The means and medians of the posterior distribution of transition-rate and root-state estimates, the MCMC effective sample size, and the highest posterior-density interval for Anura, Cuadata and Gymnophiona are presented in Supplementary Tables 10–12. For each of the three groups, the most aquatic mode was estimated to be the most probably root state (Supplementary Fig. 9), i.e., fully aquatic for Anura and Caudata, and semi-terrestrial for Gymnophiona.

### State-dependent diversification

Potential effects of life history on diversification rates were investigated using extensions of the State-dependent Speciation and Extinction (SSE) framework^43^. Specifically, we used Multistate Hidden State Speciation and Extinction (MuHiSSE) models from the R package hisse v1.9.6^45^ and Several Examined and Concealed States-dependent Speciation and Extinction (SecSSE) models from the R package secsse v2.1.7^46^ (from here on “hisse” and “secsse” models). Like corHMM, these packages allow rates to vary within each observed state by including hidden/concealed states. This approach has the advantage of being able to distinguish between rate variation that is directly attributable to the observed traits and rate variation related to unobserved traits. The hisse package implements multi-state traits through allowing a combination of two binary traits, whereas secsse has no theoretical limits on the number of discrete character states. We therefore used hisse to test whether changes in life cycle (i.e., loss of either the larval or adult life stage) were associated with a diversification-rate shift. We used secsse to test whether diversification-rate shifts were associated with any of the six reproductive modes examined here.

Each amphibian clade was analyzed separately. For the hisse analyses, the available trait states were: 00: no larval and no adult stage (an impossibility); 01: no larval stage but an adult stage (direct development and live-bearing); 10: a larval stage but no adult stage (paedomorphism); and 11: a larval stage and an adult stage (all forms of biphasic development). As 00 is an impossibility, all parameters associated with this state were fixed to 0. Similarly, no paedomorphic species (no adult stage) exist in Anura and Gymnophiona, and so this state was also fixed to be 0 for these clades. For the secsse analysis, models for Anura and Caudata estimated diversification rates for five states: (1) aquatic, (2) semi-terrestrial, (3) direct developing, and (4) live-bearing for both groups, along with fully-terrestrial for Anura (5) and paedomorphism for Caudata (5). Models for Gymnophiona estimated rates for three states (semi-terrestrial, direct development, and live-bearing).

For both analyses (hisse and secsse), and each of the three amphibian clades, we tested three scenarios: (1) constant diversification (CD models), where speciation and extinction parameters are fixed to be the same across all states. (2) Variable diversification rates across the observed traits, with or without hidden traits (Reproductive-mode Dependent Diversification; RmDD models). (3) Variable diversification, but only across hidden states (Reproductive-mode Independent Diversification models: RmID). Scenarios two and three with variable rates were repeated with up to three hidden states (four rate categories) for the hisse analysis. For the secsse analysis, the number of parameters for hidden-state models was exceedingly large and thus only one hidden state (two rate categories) could be tested. For each clade, these variations resulted in a total of eight hisse models and four secsse models. For both analyses, we ranked models according to their performance based on AIC and AICw to compare the fit of the three diversification scenarios. For the best-performing hisse models, we estimated net diversification rates (speciation minus extinction) for tips (i.e., extant species) and plotted these as distributions of net diversification rates per trait state using the R package gghisse (https://github.com/discindo/gghisse).

For the hisse analysis, the transition rates between states were not restricted. For the secsse analysis, a full transition-rate matrix would result in an extremely large number of parameters to estimate. We therefore restricted these latter matrices to allow only transitions permitted in the best-performing corHMM model for each clade (see Results). For both analyses, transitions between observed trait states and hidden states were restricted to reduce model complexity. Specifically, dual transitions (e.g., reproductive mode state 1 with hidden state A transitioning to reproductive mode state 2 with hidden state B) were not permitted and all transition rates between hidden traits were restricted to be the same. Secsse models were also repeated with the extinction rate fixed to zero across all character states, to test sensitivity to extinction-rate estimates. This did not significantly change the order of the model performances (see Supplementary Table 6) and is therefore not discussed in the results. Hisse does not estimate speciation and extinction rates directly, but rather species turnover (speciation + extinction rates) and extinction fraction (speciation/extinction rates), which are then used to calculate net diversification rates.

For the hisse analyses, the root state probabilities were fixed to be 11 (biphasic) for all three clades, and species not represented in the tree were accounted for via state-specific sampling fractions (Anura: 01 = 38.8%, 11 = 51.7%; Caudata: 01 = 74.8%, 10 = 80.1%, 11 = 71.6%; Gymnophiona: 00 = 32.1% 11 = 25.3%). Thus, for example, the value of 74.8% for Caudata means that 74.8% of the species with state 01 are represented in the tree, based on our sampling of 99.5% of caudates with data on reproductive mode. For the secsse analysis, the root states were fixed to be aquatic biphasic for Anura and Caudata and semi-terrestrial biphasic for Gymnophiona and state-specific sampling fractions were again used to account for sampling biases (Anura: aquatic = 52.8%, semi-terrestrial = 56.5%, terrestrial = 52.2%, direct development = 39.0%, live-bearing = 25%; Caudata: aquatic = 72.2%, semi-terrestrial = 75.7%, direct development = 74.2%, live-bearing = 92.9%, paedomorphism = 81.0%; Gymnophiona: semi-terrestrial = 25.3%, direct development = 45.6%, live-bearing = 38.7%).

Given that the analyses can be sensitive to starting values for parameters^104^, we ran each hisse model with 10 different starting values. These starting values were drawn from a normal distribution centered on the log initial starting value, which was generated using the starting.point.generator() function for hisse, and a standard deviation of 1 (following^104^). Each secsse model was run with three different starting values, once with optimized starting values for speciation and extinction generated with the bd_ML() function from the DDD package v5.0^105^, and then with double and half these rates. The starting values for transition rates were a fifth of the speciation rates. For each model, only the try with the best starting values (based on the model’s log likelihood) was kept for the final model comparisons. We checked that models had converged and that nested models had log likelihoods that were lower or the same as more complete models.

### Reporting summary

Further information on research design is available in the Nature Portfolio Reporting Summary linked to this article.

## Supplementary information

Supplementary Information

Description of Additional Supplementary Files

Supplementary Data 1

Supplementary Data 2

Supplementary Data 3

Supplementary Data 4

Reporting Summary

## Data Availability

Supplementary Data 1 contains the reproductive mode data for each species and supporting references. Supplementary Data 2 rate estimates for the best peforming hisse and secsse models. Supplementary Data 3 contains the taxonomic changes made to the phylogeny. Supplenetary Data 4 contains the phylogeny used for the comparative analyses. The taxonomy used follows that of the Amphibian Species of the World v6.0 (https://amphibiansoftheworld.amnh.org/). Source data are provided with this paper.

## Source data

Source Data

## Code availability

The R code and BayesTraits commands used in this study are deposited on Zenodo (10.5281/zenodo.7215080).
