# Supplementary material for: The evolution of reproductive modes and life cycles in amphibians
Source: Nat Commun. 2022 Nov 17;13:7039. doi: 10.1038/s41467-022-34474-4 (PMC9672123; doi:10.1038/s41467-022-34474-4)
Supplement: Supplementary file 1 — Supplementary Information [file 41467_2022_34474_MOESM1_ESM.pdf]

Supplementary Information

Supplementary Tables

Supplementary Table 1: Results for corHMM trait evolution model fitting (Anura).
Supplementary Table 2: Results for corHMM trait evolution model fitting (Caudata).
Supplementary Table 3: Results for corHMM trait evolution model fitting (Gymnophiona).
Supplementary Table 4: Results for corHMM trait evolution model fitting (Anura with 4-state coding).
Supplementary Table 5: Results for corHMM trait evolution model fitting (without species with no or inferred reproductive modes).
Supplementary Table 6: Results for secsse diversification rate model fitting, where extinction is fixed to be 0.
Supplementary Table 7: Comparison of reproductive mode coding to that of Duellman and Trueb 1994.
Supplementary Table 8: Effect of root prior on corHMM models. Performance measured using Akaike Information Criterion (AIC), when implementing different transition rate restrictions (ER= equal rates; SYM=symmetric rates; ARD=all rates different) and root priors (MaddFitz and Yang), with or without hidden states (HMM=Hidden Markov Model).
Supplementary Table 9: Log Marginal Likelihood (log ML) estimates using the standard versus covarion BayesTraits Multistate algorithms with reverse jump MCMC and a stepping stone sampler, for Anura, Caudata and Gymnophiona.
Supplementary Table 10: Posterior transition rate metrics for Anura, estimated using the covarion BayesTraits Multistate algorithms with reverse jump MCMC. Tabulated are the mean and median transition rates for each transition (where states are Aquatic [A], Semi-terrestrial [S], Terrestrial [T], Direct development [D] and Live-bearing [L]) as well as the percentage any given transition was not fixed to be zero in the posterior (i.e. how frequently is a given transition represented in the posterior) and the Effective Sample Size (ESS) + Highest probability Density interval (HPD) for the mean rate. Transitions are ranked by how frequently they were not zero.
Supplementary Table 11: Posterior transition rate metrics for Caudata, estimated using the covarion BayesTraits Multistate algorithms with reverse jump MCMC. Tabulated are the mean and

median transition rates for each transition (where states are Aquatic [A], Semi-terrestrial [S], Direct development [D], Live-bearing [L], and Paedomorphism[P]), as well as the percentage any given transition was not fixed to be zero in the posterior (i.e. how frequently is a given transition represented in the posterior) and the Effective Sample Size (ESS) + Highest probability Density interval (HPD) for the mean rate. Transitions are ranked by how frequently they were not zero.

Supplementary Table 12: Posterior transition rate metrics for Gymnophiona, estimated using the covarion BayesTraits Multistate algorithms with reverse jump MCMC. Tabulated are the mean and median transition rates for each transition (where states are Semi-terrestrial [S], Direct development [D] and Live-bearing [L]) as well as the percentage any given transition was not fixed to be zero in the posterior (i.e. how frequently is a given transition represented in the posterior) and the Effective Sample Size (ESS) + Highest probability Density interval (HPD) for the mean rate. Transitions are ranked by how frequently they were not zero.

Supplementary Figures

Supplementary Figure 1: Ancestral state estimates of reproductive modes for Anura based on best-fitting corHMM model parameters and the joint estimation method. States are mapped onto the phylogeny with nodes and associated upstream branches coloured based on the estimated node state. Blue = aquatic; dark green = semi-terrestrial; brown = terrestrial; light green = direct development; red = live-bearing.

Supplementary Figure 2: Ancestral state estimates of reproductive modes for Caudata based on best-fitting corHMM model parameters and the joint estimation method. States are mapped onto the phylogeny with nodes and associated upstream branches coloured based on the estimated node state. Blue = aquatic; dark green = semi-terrestrial; light green = direct development; red = live-bearing; yellow = paedomorphism.

Supplementary Figure 3: Ancestral state estimates of reproductive modes for Gymnophiona, based on best-fitting corHMM model parameters and the joint estimation method. States are mapped onto the phylogeny with nodes and associated upstream branches coloured based on the estimated node state. Dark green = semi-terrestrial; light green = direct development; red = live-bearing.

Supplementary Figure 4: Ancestral state estimates of reproductive modes for Anura based on best-fitting corHMM model parameters and the stochastic character mapping method. States are mapped onto the phylogeny with nodes depicting pie charts of posterior probabilities of node states from 1000 simulations. Upstream branches are coloured based on the largest proportion of the estimated node state. Blue = aquatic; dark green = semi-terrestrial; brown = terrestrial; light green = direct development; red = live-bearing.

Supplementary Figure 5: Ancestral state estimates of reproductive modes for Caudata based on best-fitting corHMM model parameters and the stochastic character mapping method. States are mapped onto the phylogeny with nodes depicting pie charts of posterior probabilities of node states from 1000 simulations. Upstream branches are coloured based on the largest proportion of the estimated node state. Blue = aquatic; dark green = semi-terrestrial; light green = direct development; red = live-bearing; yellow = paedomorphism.

Supplementary Figure 6: Ancestral state estimates of reproductive modes for Gymnophiona based on best-fitting corHMM model parameters and the stochastic character mapping method. States are mapped onto the phylogeny with nodes depicting pie charts of posterior probabilities of node states from 1000 simulations. Upstream branches are coloured based on the largest proportion of the estimated node state. Dark green = semi-terrestrial; light green = direct development; red = live-bearing.

Supplementary Figure 7: Marginal probabilities of reproductive mode of the most recent common ancestor of all amphibians and for Anura, Caudata and Gymnophiona. Probabilities were estimated using transition rates estimated from different models (er= equal rates; sym=symmetric rates; ard=all rates different), without and with hidden states (HMM=hidden Markov model) and two different root prior methods, MaddFitz and Yang, using corHMM. Blue = aquatic; dark green = semi-terrestrial; brown = terrestrial; light green = direct development; red = live-bearing, yellow = paedomorphism.

Supplementary Figure 8: Representation (% of the posterior) of the 25 most frequent models sampled by the covarion BayesTraits Multistate algorithms with reverse jump MCMC for Anura, Caudata and Gymnophiona.

Supplementary Figure 9: Pie charts showing the mean posterior probabilities of root states per amphibian group (Anura, Caudata and Gymnophiona) as estimated using the covarion BayesTraits Multistate algorithms with reverse jump MCMC. Blue = aquatic; dark green = semi-terrestrial; brown = terrestrial; light green = direct development; red = live-bearing, yellow = paedomorphism.

Supplementary Notes

Supplementary Note 1: Root state estimations for the Amphibia, using corHMM

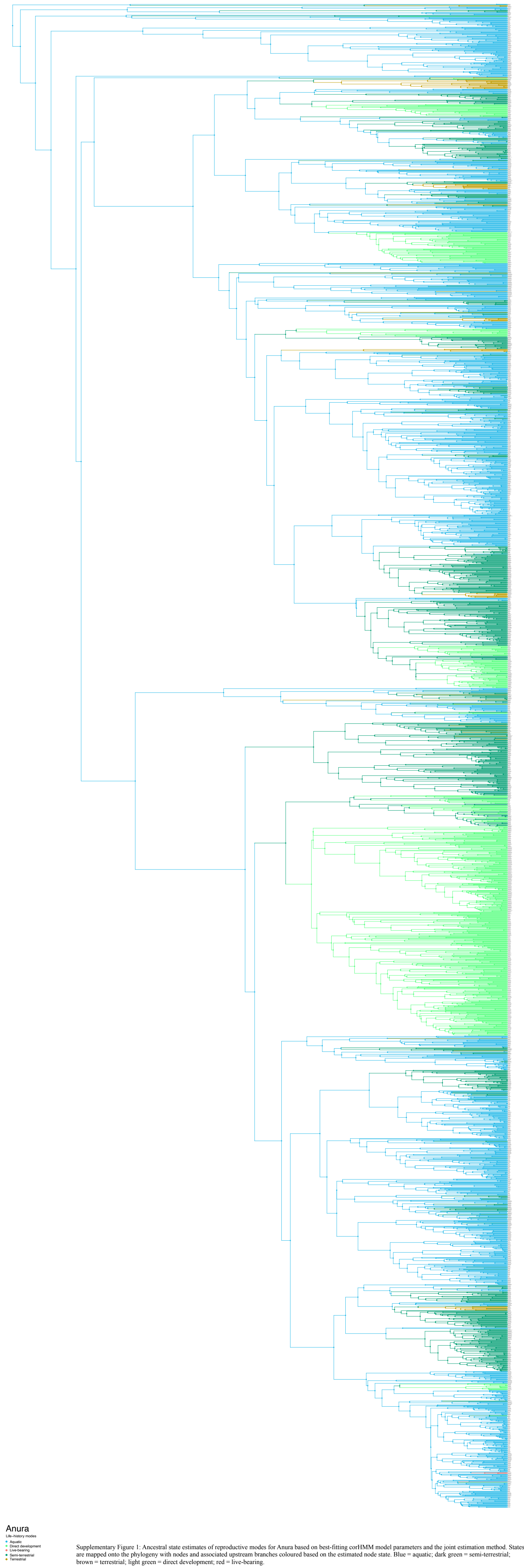

Supplementary Figure 1: Ancestral state estimates of reproductive modes for Anura based on best-fitting corHMM model parameters and the joint estimation method. States are mapped onto the phylogeny with nodes and associated upstream branches coloured based on the estimated node state. Blue = aquatic; dark green = semi-terrestrial; brown = terrestrial; light green = direct development; red = live-bearing.

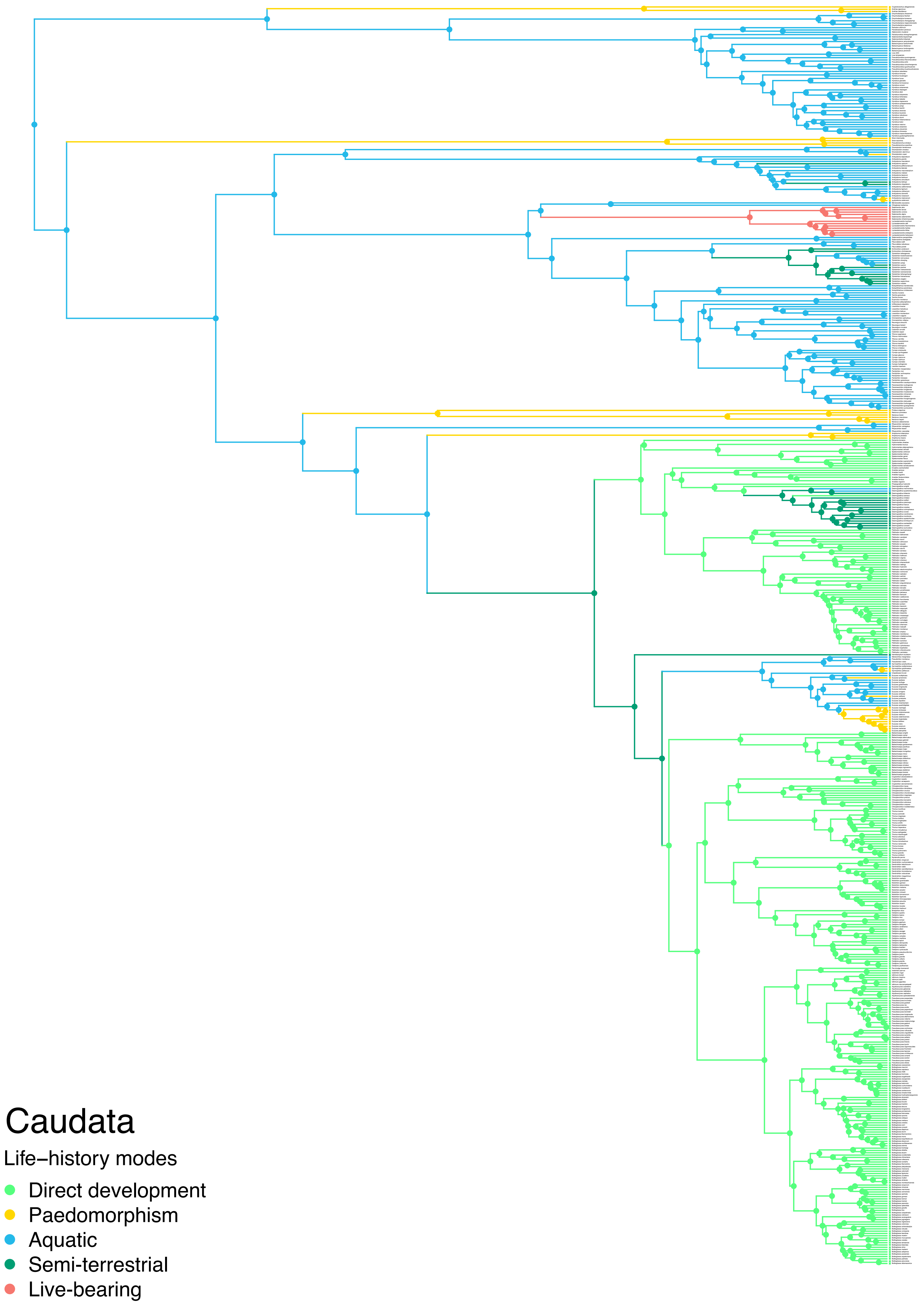

Supplementary Figure 2: Ancestral state estimates of reproductive modes for Caudata based on best-fitting corHMM model parameters and the joint estimation method. States are mapped onto the phylogeny with nodes and associated upstream branches coloured based on the estimated node state. Blue = aquatic; dark green = semi-terrestrial; light green = direct development; red = live-bearing; yellow = paedomorphism.

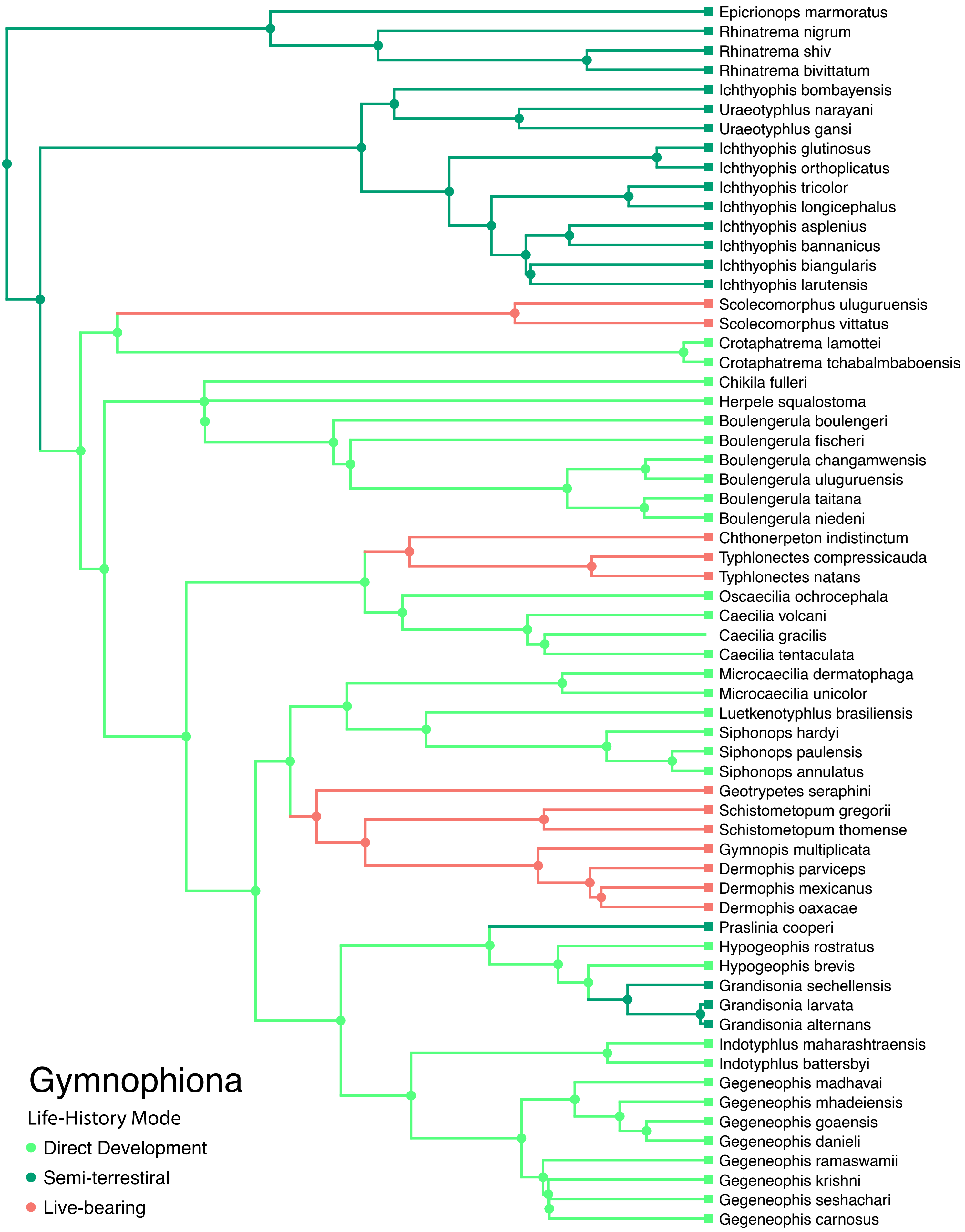

Supplementary Figure 3: Ancestral state estimates of reproductive modes for Gymnophiona, based on best-fitting corHMM model parameters and the joint estimation method. States are mapped onto the phylogeny with nodes and associated upstream branches coloured based on the estimated node state. Dark green = semi-terrestrial; light green = direct development; red = live-bearing.

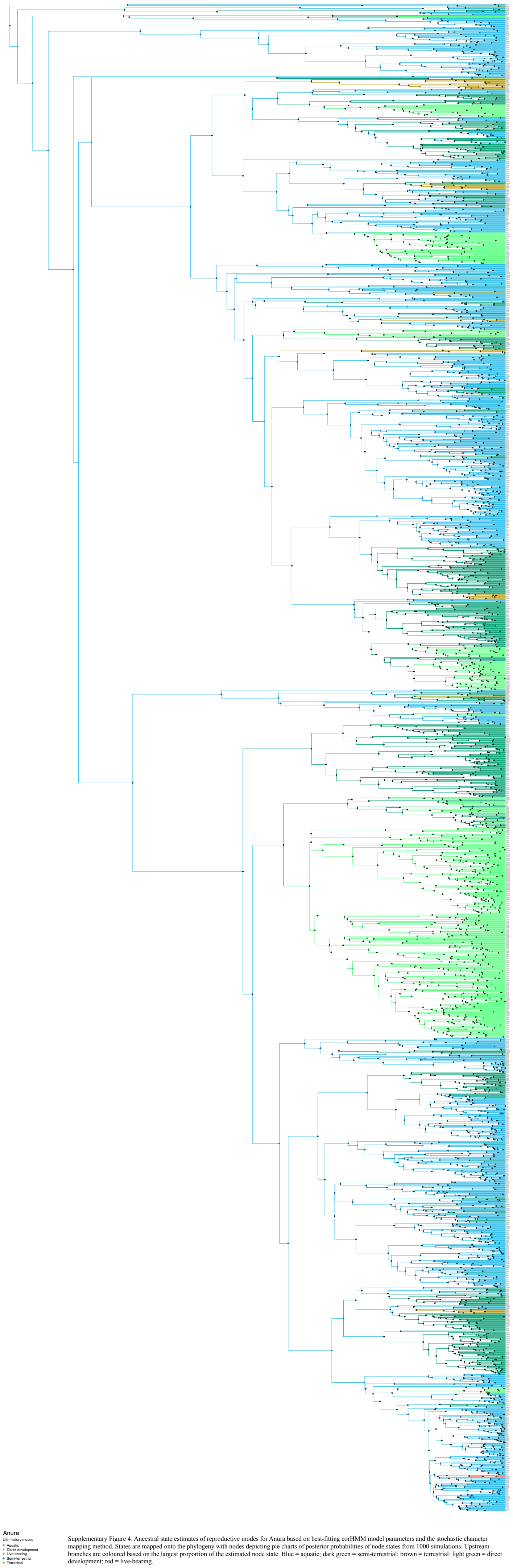

Supplementary Figure 4: Ancestral state estimates of reproductive modes for Anura based on best-fitting corHMM model parameters and the stochastic character mapping method. States are mapped onto the phylogeny with nodes depicting pie charts of posterior probabilities of node states from 1000 simulations. Upstream branches are coloured based on the largest proportion of the estimated node state. Blue = aquatic; dark green = semi-terrestrial; brown = terrestrial; light green = direct development; red = live-bearing.

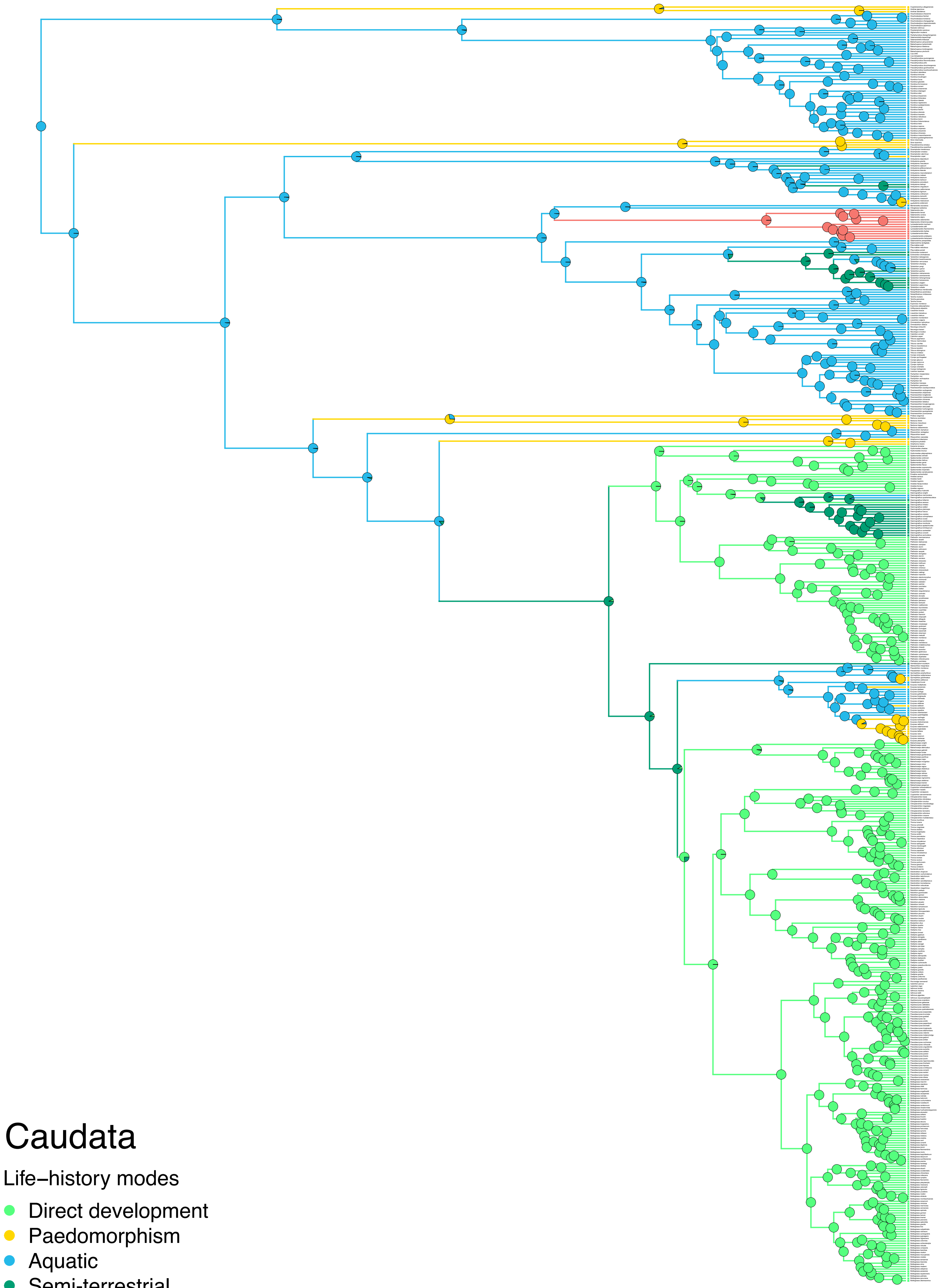

Supplementary Figure 5: Ancestral state estimates of reproductive modes for Caudata based on best-fitting corHMM model parameters and the stochastic character mapping method. States are mapped onto the phylogeny with nodes depicting pie charts of posterior probabilities of node states from 1000 simulations. Upstream branches are coloured based on the largest proportion of the estimated node state. Blue = aquatic; dark green = semi-terrestrial; light green = direct development; red = live-bearing; yellow = paedomorphism.

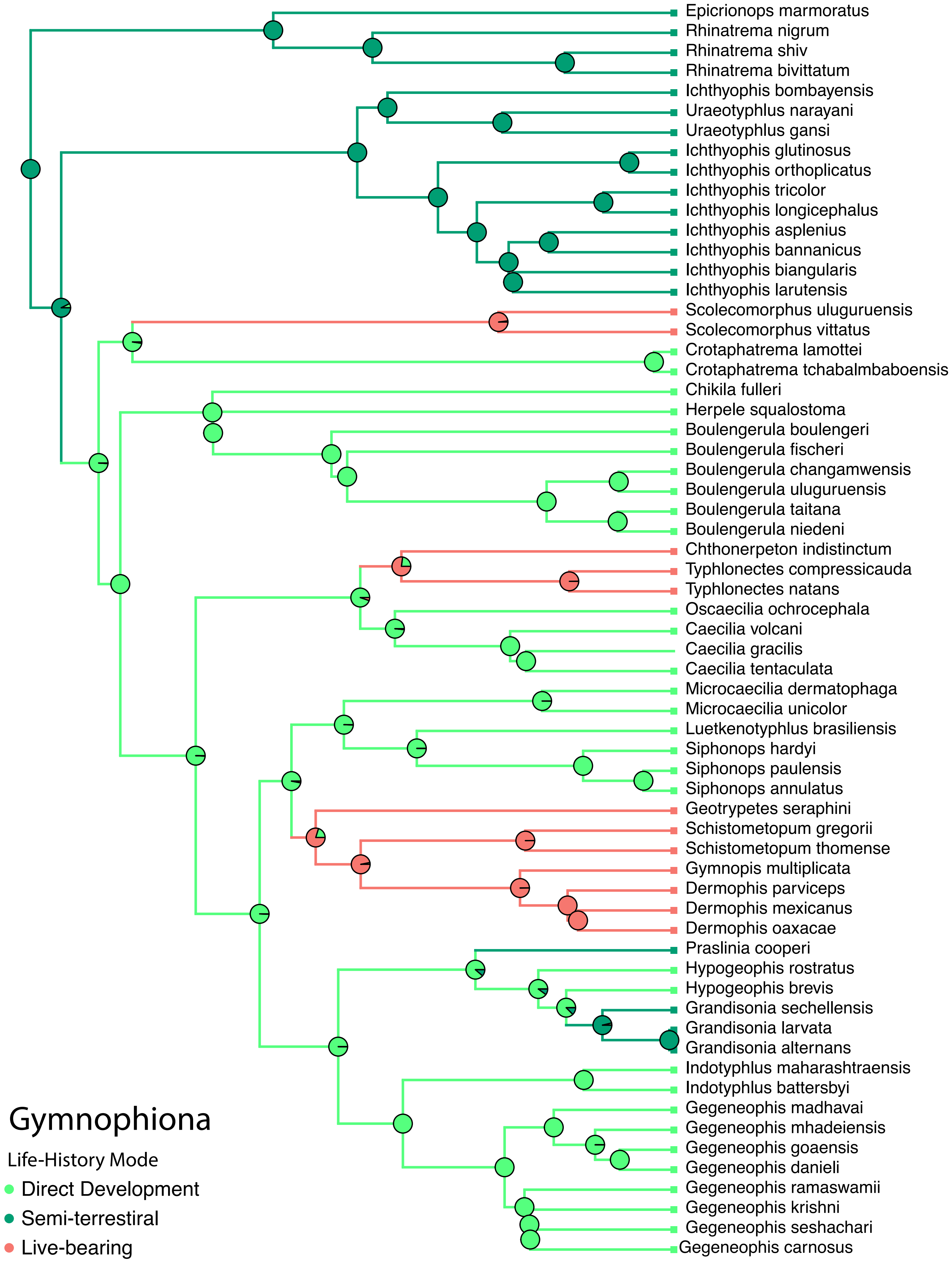

Supplementary Figure 6: Ancestral state estimates of reproductive modes for Gymnophiona based on best-fitting corHMM model parameters and the stochastic character mapping method. States are mapped onto the phylogeny with nodes depicting pie charts of posterior probabilities of node states from 1000 simulations. Upstream branches are coloured based on the largest proportion of the estimated node state. Dark green = semi-terrestrial; light green = direct development; red = live-bearing.

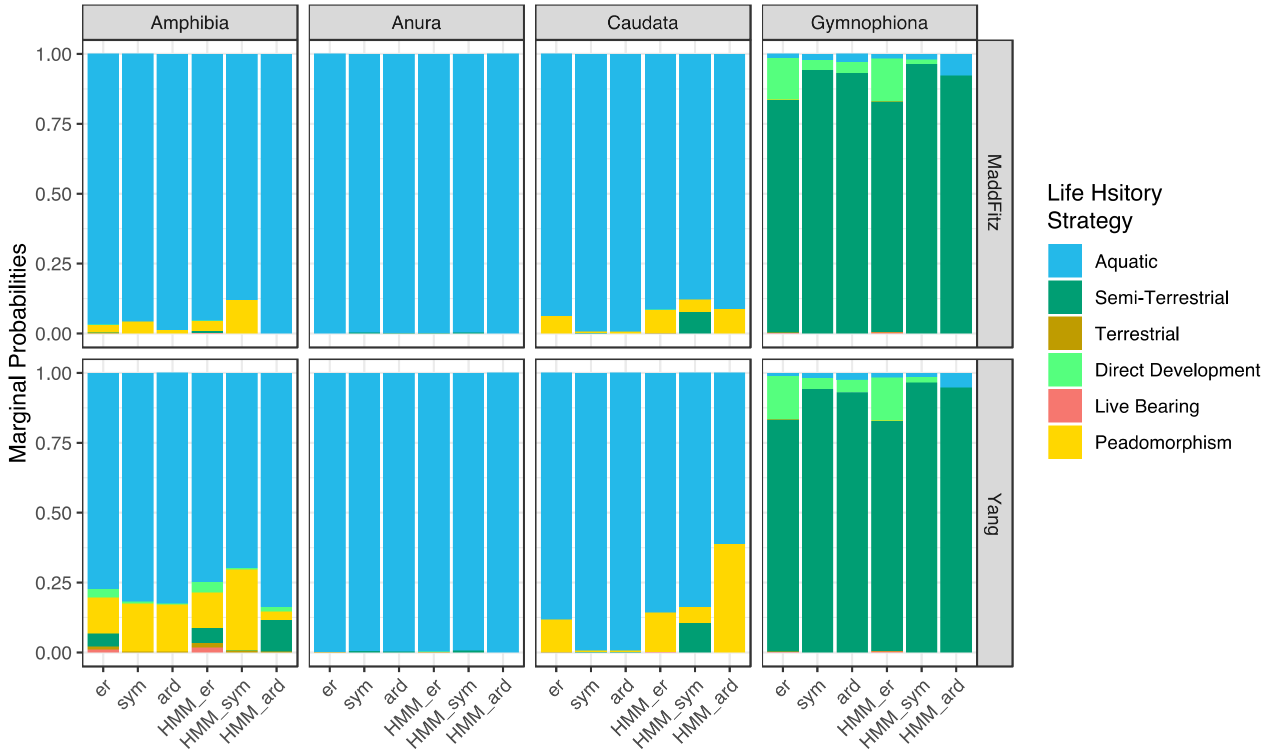

Supplementary Figure 7: Marginal probabilities of reproductive mode of the most recent common ancestor of all amphibians and for Anura, Caudata and Gymnophiona. Probabilities were estimated using transition rates estimated from different models (er= equal rates; sym=symmetric rates; ard=all rates different), without and with hidden states (HMM=hidden Markov model) and two different root prior methods, MaddFitz and Yang, using corHMM. Blue = aquatic; dark green = semi-terrestrial; brown = terrestrial; light green = direct development; red = live-bearing, yellow = paedomorphism.

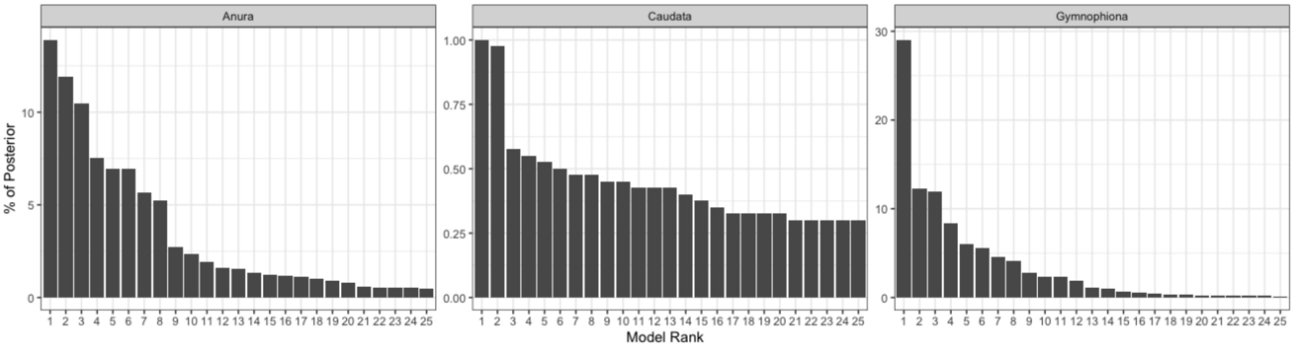

Supplementary Figure 8: Representation (% of the posterior) of the 25 most frequent models sampled by the covarion BayesTraits Multistate algorithms with reverse jump MCMC for Anura, Caudata and Gymnophiona.

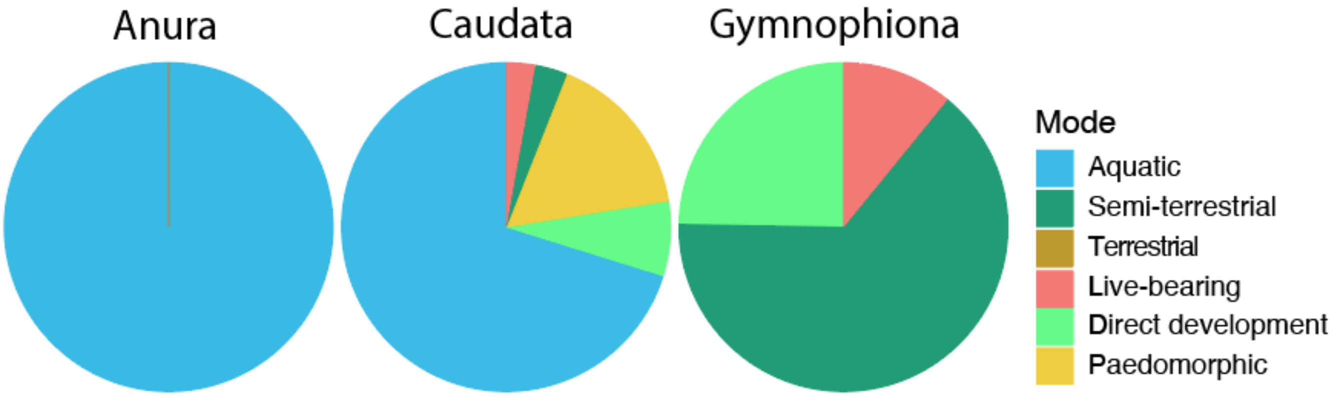

Supplementary Figure 9: Pie charts showing the mean posterior probabilities of root states per amphibian group (Anura, Caudata and Gymnophiona) as estimated using the covarion BayesTraits Multistate algorithms with reverse jump MCMC. Blue = aquatic; dark green = semi-terrestrial; brown = terrestrial; light green = direct development; red = live-bearing, yellow = paedomorphism.

Supplementary Table 1. corHMM model fits for Anura. Model names refer to those described in Figure 2. For each model, rate class refers to whether models included observed only (Mk) or observed plus one set of hidden states (HMM). Each model was run with either no rate restrictions (ARD), rates fixed to be all equal (ER) or to be symmetrical (SYM). Model performance metrics given are: log likelihood, Akaike Information Criterion (AIC), the difference in AIC score between the best model and the model being compared (dAIC) and Akaike Weights, a measure of the conditional probabilities for each model (weights add up to 1).

Model Name	Transitions	Rate Class	Log Likelihood	AIC	dAIC	Akaike Weights
**Semi-sequential V1**	ARD	HMM	-667.664	1361.328	0.000	0.732
**Semi-sequential V2**	ARD	HMM	-668.674	1363.347	2.019	0.267
**Null**	ARD	HMM	-660.185	1374.370	13.042	0.001
**Semi-sequential V3**	ARD	HMM	-676.836	1379.672	18.343	0.000
**Non-sequential**	ARD	HMM	-684.275	1394.550	33.222	0.000
**Null**	SYM	HMM	-688.729	1419.459	58.130	0.000
**Semi-sequential V1**	SYM	HMM	-700.843	1419.686	58.358	0.000
**Semi-sequential V2**	SYM	HMM	-705.200	1428.399	67.071	0.000
**Semi-sequential V3**	SYM	HMM	-712.204	1442.408	81.079	0.000
**Null**	ARD	Mk	-714.653	1455.306	93.977	0.000
**Null**	SYM	Mk	-719.684	1459.368	98.040	0.000
**Semi-sequential V3**	ER	HMM	-728.355	1462.711	101.382	0.000
**Semi-sequential V2**	ER	HMM	-728.790	1463.580	102.252	0.000
**Semi-sequential V1**	ARD	Mk	-729.936	1471.873	110.544	0.000
**Semi-sequential V1**	SYM	Mk	-735.069	1478.137	116.809	0.000
**Semi-sequential V2**	ARD	Mk	-733.921	1479.842	118.514	0.000
**Non-sequential**	SYM	HMM	-734.232	1486.464	125.136	0.000
**Semi-sequential V2**	SYM	Mk	-739.280	1486.561	125.232	0.000
**Sequential**	ARD	HMM	-734.602	1491.204	129.875	0.000
**Semi-sequential V3**	ARD	Mk	-741.880	1495.760	134.432	0.000
**Semi-sequential V3**	SYM	Mk	-747.508	1503.016	141.687	0.000
**Semi-sequential V1**	ER	HMM	-756.174	1518.347	157.019	0.000
**Semi-sequential V2**	ER	Mk	-771.555	1545.110	183.781	0.000
**Sequential**	SYM	HMM	-766.812	1551.623	190.295	0.000
**Semi-sequential V3**	ER	Mk	-777.249	1556.498	195.169	0.000
**Semi-sequential V1**	ER	Mk	-786.500	1575.000	213.672	0.000
**Null**	ER	HMM	-785.049	1576.097	214.769	0.000
**Sequential**	ER	HMM	-793.774	1593.548	232.219	0.000
**Null**	ER	Mk	-814.303	1630.606	269.278	0.000
**Non-sequential**	ER	HMM	-813.797	1633.593	272.265	0.000
**Non-sequential**	SYM	Mk	-813.102	1634.204	272.876	0.000
**Non-sequential**	ARD	Mk	-812.917	1637.834	276.506	0.000
**Sequential**	ARD	Mk	-834.604	1679.208	317.879	0.000
**Sequential**	SYM	Mk	-838.276	1684.553	323.224	0.000
**Sequential**	ER	Mk	-864.860	1731.720	370.391	0.000
**Non-sequential**	ER	Mk	-885.517	1773.033	411.705	0.000

Supplementary Table 2. coHMM model fits for Caudata. Model names refer to those described in Figure 2. For each model, rate class refers to whether models included observed only (Mk) or observed plus one set of hidden states (HMM). Model performance metrics given are: log likelihood, Akaike Information Criterion (AIC), the difference in AIC score between the best model and the model being compared (dAIC) and Akaike Weights, a measure of the conditional probabilities for each model (weights add up to 1).

Model Name	Transitions	Rate Class	Log Likelihood	AIC	dAIC	Akaike Weights
**Semi-sequential V1**	ARD	Mk	-109.430	232.861	0.000	0.454
**Semi-sequential V2**	ER	HMM	-114.032	234.064	1.203	0.249
**Semi-sequential V2**	ARD	Mk	-110.874	235.747	2.886	0.107
**Semi-sequential V1**	SYM	HMM	-108.941	235.881	3.020	0.100
**Non-sequential**	SYM	HMM	-110.256	238.512	5.651	0.027
**Semi-sequential V1**	ER	HMM	-116.732	239.463	6.602	0.017
**Sequential**	ER	HMM	-117.079	240.158	7.298	0.012
**Semi-sequential V2**	SYM	HMM	-111.175	240.351	7.490	0.011
**Semi-sequential V2**	ARD	HMM	-105.066	240.132	7.271	0.012
**Non-sequential**	ARD	HMM	-105.868	241.736	8.875	0.005
**Sequential**	ARD	Mk	-114.358	242.715	9.854	0.003
**Non-sequential**	ER	HMM	-119.783	245.565	12.705	0.001
**Sequential**	ARD	HMM	-107.662	245.324	12.463	0.001
**Semi-sequential V2**	ER	Mk	-123.282	248.564	15.704	0.000
**Semi-sequential V1**	ARD	HMM	-108.988	247.977	15.116	0.000
**Sequential**	SYM	HMM	-115.284	248.568	15.707	0.000
**Semi-sequential V1**	SYM	Mk	-120.791	249.583	16.722	0.000
**Non-sequential**	ARD	Mk	-117.979	249.957	17.096	0.000
**Semi-sequential V2**	SYM	Mk	-121.626	251.253	18.392	0.000
**Null**	ARD	Mk	-109.430	250.861	18.000	0.000
**Non-sequential**	SYM	Mk	-122.263	252.527	19.666	0.000
**Semi-sequential V1**	ER	Mk	-126.694	255.387	22.526	0.000
**Null**	ER	HMM	-125.149	256.299	23.438	0.000
**Null**	SYM	Mk	-118.721	257.442	24.581	0.000
**Null**	SYM	HMM	-108.261	258.523	25.662	0.000
**Non-sequential**	ER	Mk	-129.644	261.288	28.427	0.000
**Sequential**	SYM	Mk	-127.103	262.206	29.345	0.000
**Sequential**	ER	Mk	-132.632	267.263	34.402	0.000
**Null**	ER	Mk	-134.830	271.660	38.799	0.000
**Null**	ARD	HMM	-100.729	267.459	34.598	0.000

Supplementary Table 3. coHMM model fits for Gymnophiona. Model names refer to those described in Figure 2. For each model, rate class refers to whether models included observed only (Mk) or observed plus one set of hidden states (HMM). Model performance metrics given are: log likelihood, Akaike Information Criterion (AIC), the difference in AIC score between the best model and the model being compared (dAIC) and Akaike Weights, a measure of the conditional probabilities for each model (weights add up to 1).

Model Name	Transitions	Rate Class	Log Likelihood	AIC	dAIC	Akaike Weights
**Sequential**	ER	Mk	-29.045	60.089	0.000	0.421
**Sequential**	SYM	Mk	-28.987	61.973	1.884	0.164
**Sequential**	ER	HMM	-28.085	62.171	2.081	0.149
**Null**	ER	Mk	-30.690	63.380	3.291	0.081
**Null**	SYM	Mk	-28.987	63.973	3.884	0.060
**Sequential**	ARD	Mk	-28.172	64.343	4.254	0.050
**Null**	ER	HMM	-29.633	65.266	5.177	0.032
**Sequential**	SYM	HMM	-28.057	66.113	6.024	0.021
**Null**	ARD	Mk	-28.172	68.343	8.254	0.007
**Semi-sequential V2**	ARD	HMM	-24.951	67.901	7.812	0.008
**Null**	SYM	HMM	-28.057	70.113	10.024	0.003
**Semi-sequential V2**	ER	HMM	-33.614	73.227	13.138	0.001
**Sequential**	ARD	HMM	-26.133	70.267	10.178	0.003
**Semi-sequential V2**	ARD	Mk	-32.517	73.034	12.945	0.001
**Semi-sequential V2**	ER	Mk	-37.387	76.774	16.685	0.000
**Semi-sequential V2**	SYM	HMM	-33.566	77.132	17.043	0.000
**Semi-sequential V2**	SYM	Mk	-37.313	78.626	18.536	0.000
**Null**	ARD	HMM	-24.951	75.901	15.812	0.000

Supplementary Table 4. coHMM model fits for Anura, where "S" and "T" trait states have been combined so that there are only four states (see methods). Model names refer to those described in Figure 2. For each model, rate class refers to whether models included observed only (Mk) or observed plus one set of hidden states (HMM). Model performance metrics given are: log likelihood, Akaike Information Criterion (AIC), the difference in AIC score between the best model and the model being compared (dAIC) and Akaike Weights, a measure of the conditional probabilities for each model (weights add up to 1).

Model Name	Transitions	Rate Class	Log Likelihood	AIC	dAIC	Akaike Weights
**Semi-sequential V1**	ARD	HMM	-557.942	1137.885	0.000	0.791
**Semi-sequential V2**	ARD	HMM	-559.288	1140.576	2.691	0.206
**Non-sequential**	ARD	HMM	-563.734	1149.468	11.583	0.002
**Null**	ARD	HMM	-556.555	1151.111	13.226	0.001
**Sequential**	ARD	HMM	-567.399	1156.797	18.912	0.000
**Null**	SYM	HMM	-586.565	1199.130	61.245	0.000
**Semi-sequential V1**	SYM	HMM	-598.646	1211.291	73.406	0.000
**Semi-sequential V2**	SYM	HMM	-602.965	1219.931	82.046	0.000
**Sequential**	SYM	HMM	-608.826	1231.652	93.767	0.000
**Non-sequential**	SYM	HMM	-609.160	1232.320	94.435	0.000
**Null**	ARD	Mk	-608.389	1234.777	96.892	0.000
**Null**	SYM	Mk	-615.590	1243.180	105.295	0.000
**Semi-sequential V1**	ARD	Mk	-620.046	1250.092	112.207	0.000
**Sequential**	ER	HMM	-624.372	1254.743	116.858	0.000
**Semi-sequential V2**	ARD	Mk	-624.161	1258.323	120.438	0.000
**Semi-sequential V1**	SYM	Mk	-626.488	1258.977	121.092	0.000
**Semi-sequential V2**	ER	HMM	-629.286	1264.571	126.686	0.000
**Semi-sequential V2**	SYM	Mk	-630.868	1267.736	129.851	0.000
**Sequential**	ARD	Mk	-632.080	1274.160	136.275	0.000
**Sequential**	SYM	Mk	-639.005	1284.010	146.125	0.000
**Semi-sequential V1**	ER	HMM	-651.113	1308.227	170.342	0.000
**Semi-sequential V2**	ER	Mk	-664.537	1331.074	193.189	0.000
**Sequential**	ER	Mk	-668.448	1338.896	201.011	0.000
**Non-sequential**	ARD	Mk	-665.076	1340.151	202.266	0.000
**Non-sequential**	SYM	Mk	-669.801	1345.602	207.717	0.000
**Semi-sequential V1**	ER	Mk	-676.849	1355.699	217.814	0.000
**Null**	ER	HMM	-680.179	1366.359	228.474	0.000
**Non-sequential**	ER	HMM	-688.119	1382.237	244.352	0.000
**Null**	ER	Mk	-707.263	1416.526	278.641	0.000
**Non-sequential**	ER	Mk	-734.995	1471.991	334.106	0.000

Supplementary Table 5a. corHMM model fits for Anura, without species with "inferred" and unknown (NA) life history coding. Model names refer to those described in Figure 2. For each model, rate class refers to whether models included observed only (Mk) or observed plus one set of hidden states (HMM). Model performance metrics given are: log likelihood, Akaike Information Criterion (AIC), the difference in AIC score between the best model and the model being compared (dAIC) and Akaike Weights, a measure of the conditional probabilities for each model (weights add up to 1).

Model Name	Transitions	Rate Class	Log Likelihood	AIC	dAIC	Akaike Weights
**Semi-sequential V1**	ARD	HMM	-582.940	1191.880	1191.987	0.999
**Semi-sequential V2**	ARD	HMM	-590.793	1207.585	1207.692	0.000
**Null**	ARD	HMM	-577.308	1208.616	1209.062	0.000
**Non-sequential**	ARD	HMM	-596.943	1219.886	1219.993	0.000
**Semi-sequential V3**	ARD	HMM	-600.437	1226.874	1226.981	0.000
**Semi-sequential V1**	SYM	HMM	-610.506	1239.011	1239.064	0.000
**Null**	SYM	HMM	-603.853	1249.707	1249.979	0.000
**Semi-sequential V2**	SYM	HMM	-619.074	1256.148	1256.201	0.000
**Null**	ARD	Mk	-624.085	1274.169	1274.276	0.000
**Semi-sequential V3**	SYM	HMM	-628.257	1274.513	1274.566	0.000
**Null**	SYM	Mk	-627.902	1275.804	1275.869	0.000
**Semi-sequential V2**	ER	HMM	-639.730	1285.459	1285.466	0.000
**Semi-sequential V1**	ARD	Mk	-638.494	1288.988	1289.012	0.000
**Semi-sequential V3**	ER	HMM	-641.731	1289.461	1289.468	0.000
**Semi-sequential V1**	SYM	Mk	-642.583	1293.165	1293.177	0.000
**Non-sequential**	SYM	HMM	-639.005	1296.010	1296.063	0.000
**Semi-sequential V2**	ARD	Mk	-645.305	1302.610	1302.635	0.000
**Semi-sequential V2**	SYM	Mk	-649.723	1307.445	1307.457	0.000
**Sequential**	ARD	HMM	-650.545	1323.091	1323.168	0.000
**Semi-sequential V3**	ARD	Mk	-655.836	1323.673	1323.697	0.000
**Semi-sequential V1**	ER	HMM	-661.487	1328.974	1328.981	0.000
**Semi-sequential V3**	SYM	Mk	-660.991	1329.981	1329.993	0.000
**Semi-sequential V2**	ER	Mk	-677.567	1357.133	1357.134	0.000
**Null**	ER	HMM	-684.553	1375.106	1375.113	0.000
**Semi-sequential V3**	ER	Mk	-686.560	1375.119	1375.120	0.000
**Sequential**	SYM	HMM	-678.689	1375.377	1375.430	0.000
**Semi-sequential V1**	ER	Mk	-688.322	1378.643	1378.644	0.000
**Sequential**	ER	HMM	-703.411	1412.821	1412.828	0.000
**Non-sequential**	SYM	Mk	-706.547	1421.094	1421.106	0.000
**Null**	ER	Mk	-710.387	1422.775	1422.776	0.000
**Non-sequential**	ARD	Mk	-706.361	1424.722	1424.746	0.000
**Non-sequential**	ER	HMM	-709.406	1424.812	1424.819	0.000
**Sequential**	ARD	Mk	-744.489	1498.978	1498.996	0.000
**Sequential**	SYM	Mk	-746.922	1501.844	1501.855	0.000
**Non-sequential**	ER	Mk	-769.244	1540.487	1540.488	0.000
**Sequential**	ER	Mk	-771.921	1545.843	1545.844	0.000

Supplementary Table 5b. corHMM model fits for Caudata, without species with "inferred" and unknown (NA) life history coding. Model names refer to those described in Figure 2. For each model, rate class refers to whether models included observed only (Mk) or observed plus one set of hidden states (HMM). Model performance metrics given are: log likelihood, Akaike Information Criterion (AIC), the difference in AIC score between the best model and the model being compared (dAIC) and Akaike Weights, a measure of the conditional probabilities for each model (weights add up to 1).

Model Name	Transitions	Rate Class	Log Likelihood	AIC	dAIC	Akaike Weights
**Semi-sequential V1**	ARD	Mk	-109.203	232.406	232.614	0.436
**Semi-sequential V2**	ER	HMM	-113.965	233.930	233.975	0.221
**Semi-sequential V2**	ARD	Mk	-110.640	235.280	235.488	0.104
**Semi-sequential V1**	ARD	HMM	-102.413	234.825	235.731	0.092
**Semi-sequential V1**	SYM	HMM	-108.865	235.731	236.066	0.078
**Non-sequential**	SYM	HMM	-110.184	238.369	238.704	0.021
**Semi-sequential V1**	ER	HMM	-116.661	239.322	239.366	0.015
**Sequential**	ER	HMM	-117.023	240.045	240.089	0.010
**Semi-sequential V2**	SYM	HMM	-111.102	240.204	240.540	0.008
**Semi-sequential V2**	ARD	HMM	-105.043	240.086	240.992	0.007
**Non-sequential**	ARD	HMM	-105.684	241.368	242.273	0.003
**Sequential**	ARD	Mk	-114.124	242.248	242.456	0.003
**Non-sequential**	ER	HMM	-119.711	245.422	245.466	0.001
**Sequential**	ARD	HMM	-107.599	245.198	246.104	0.001
**Semi-sequential V2**	ER	Mk	-123.061	248.121	248.129	0.000
**Sequential**	SYM	HMM	-115.211	248.423	248.759	0.000
**Semi-sequential V1**	SYM	Mk	-120.514	249.029	249.102	0.000
**Non-sequential**	ARD	Mk	-117.692	249.384	249.593	0.000
**Semi-sequential V2**	SYM	Mk	-121.347	250.695	250.769	0.000
**Null**	ARD	Mk	-109.203	250.406	251.434	0.000
**Non-sequential**	SYM	Mk	-121.972	251.944	252.018	0.000
**Semi-sequential V1**	ER	Mk	-126.443	254.885	254.893	0.000
**Null**	ER	HMM	-125.094	256.188	256.233	0.000
**Null**	SYM	Mk	-118.460	256.919	257.330	0.000
**Null**	SYM	HMM	-108.189	258.379	260.142	0.000
**Non-sequential**	ER	Mk	-129.382	260.763	260.771	0.000
**Sequential**	SYM	Mk	-126.822	261.645	261.719	0.000
**Sequential**	ER	Mk	-132.447	266.895	266.902	0.000
**Null**	ER	Mk	-134.684	271.369	271.376	0.000
**Null**	ARD	HMM	-100.553	267.106	271.489	0.000

Supplementary Table 5c. corHMM model fits for Gymnophiona, without species with “inferred” and unknown (“NA”) life history coding. Model names refer to those described in Figure 2. For each model, rate class refers to whether models included observed only (Mk) or observed plus one set of hidden states (HMM). Model performance metrics given are: log likelihood, Akaike Information Criterion (AIC), the difference in AIC score between the best model and the model being compared (dAIC) and Akaike Weights, a measure of the conditional probabilities for each model (weights add up to 1).

Model Name	Transitions	Rate Class	Log Likelihood	AIC	dAIC	Akaike Weights
**Sequential**	ER	Mk	-21.471	44.941	45.007	0.365
**Sequential**	SYM	Mk	-21.253	46.506	46.706	0.156
**Null**	ER	Mk	-22.502	47.004	47.069	0.130
**Sequential**	ER	HMM	-20.436	46.872	47.278	0.117
**Sequential**	ARD	Mk	-19.953	47.907	48.596	0.061
**Null**	SYM	Mk	-21.253	48.506	48.913	0.052
**Null**	ER	HMM	-21.278	48.555	48.962	0.050
**Semi-sequential V2**	ER	Mk	-24.652	51.304	51.370	0.015
**Sequential**	SYM	HMM	-20.281	50.561	51.614	0.013
**Semi-sequential V2**	ER	HMM	-22.629	51.258	51.664	0.013
**Semi-sequential V2**	ARD	Mk	-21.504	51.008	51.698	0.013
**Null**	ARD	Mk	-19.933	51.867	53.367	0.006
**Semi-sequential V2**	SYM	Mk	-24.594	53.187	53.387	0.006
**Semi-sequential V2**	SYM	HMM	-22.531	55.063	56.115	0.001
**Null**	SYM	HMM	-20.281	54.561	56.598	0.001
**Semi-sequential V2**	ARD	HMM	-17.667	53.335	56.731	0.001
**Sequential**	ARD	HMM	-18.813	55.626	59.022	0.000
**Null**	ARD	HMM	-17.667	61.335	68.764	0.000

Supplementary Table 6: Ranking of model performance of state dependent speciation and extinction 'secsse' models for the three major groups of amphibians: Anura, Caudata and Gymnophiona, with extinction rates set to zero. Models fall into three categories: Constant diversification (CD), Reproductive-mode Dependent Diversification (RmDD; state-associated rate shifts, with or without hidden traits), Reproductive mode Independent Diversification (RmID; rate shifts only associated with states of hidden traits, not observed reproductive modes). RmDD models can have different numbers of hidden traits, resulting in different numbers of rate categories. Model performance metrics given are: Log likelihood (lnL), Akaike information criterion (AIC), the difference in AIC score between the best model and the model being compared (dAIC), and Akaike Weights (AICw), a measure of the conditional probabilities for each model (weights add up to 1).

Diversification Rate Model	No. Rate Categories	lnL	AIC	dAIC	AICw
**Anura**					
RmID (mu=0)	2	-14598.181	29214.362	0.000	0.934
RmDD (mu=0)	2	-14586.825	29219.651	5.288	0.066
RmDD (mu=0)	1	-14797.023	29616.045	401.683	0.000
CD (mu=0)	1	-14804.711	29623.421	409.059	0.000
**Caudata**					
RmDD (mu=0)	2	-2093.317	4236.634	0.000	1.000
RmID (mu=0)	2	-2125.950	4271.899	35.266	0.000
RmDD (mu=0)	1	-2148.263	4320.525	83.892	0.000
CD (mu=0)	1	-2198.240	4412.480	175.847	0.000
**Gymnophiona**					
CD (mu=0)	1	-333.016	676.033	0.000	0.746
RmDD (mu=0)	1	-332.678	679.356	3.323	0.142
RmID (mu=0)	2	-332.909	679.818	3.785	0.112
RmDD (mu=0)	2	-330.784	691.568	15.535	0.000

Supplementary Table 7. Relationship of reproductive mode and life-cycle coding used in this manuscript compared to the categorization used by Duellman and Trueb[1].

Coding	Eggs	Larvae	Bi-/Uni-phasic	Duellman and Trueb Coding
Aquatic	Aquatic	Aquatic	Biphasic	1,2,3,4,5,6
Semi-Terrestrial	Terrestrial/Arboreal/Fossorial, Terrestrial or aquatic foam nests, internal, in specialized brooding structures such as pouches or vocal sacs.	Fully or semi aquatic (living in splash zones or films of water)	Biphasic	8,9,10,12,13,14,18,19, 21,23,24,25,26
Terrestrial	Terrestrial/Arboreal/Fossorial, Terrestrial or aquatic foam nests, internal, in specialized brooding structures such as pouches or vocal sacs.	Complete development in terrestrial nests (foam or otherwise), or in specialized brooding structures such as pouches or vocal sacs.	Biphasic	15,16,22
Direct Development	Terrestrial or in specialized brooding pouches (e.g. Pipa pipa)	No free-living larva	Uniphasic – direct developing	11,17,20,27
Live-bearing	No eggs, or eggs inside the womb of the female	Live birth at late larval stage or post metamorphosis	Uniphasic – direct developing	28,29
Paedomorphy	Aquatic or Terrestrial (although the latter is extremely rare)	Aquatic, reproductive stage (obligatory)	Uniphasic – paedomorphic	Not included

References

1. Duellman, W. E. & Trueb, L. *Biology of Amphibians*. (Johns Hopkins University Press, 1994).

Supplementary Table 8: Effect of root prior on corHMM models. Performance measured using Akaike Information Criterion (AIC), when implementing different transition rate restrictions (ER= equal rates; SYM=symmetric rates; ARD=all rates different) and root priors (MaddFitz and Yang), with or without hidden states (HMM=Hidden Markov Model).

Transition Rate Model	*Root Prior*	*Hidden states*	*Log Likelihood*	*AIC*
ARD	MaddFitz	HMM	-830.636	1735.271
ARD	Yang	HMM	-835.386	1744.772
SYM	MaddFitz	HMM	-862.521	1787.042
SYM	Yang	HMM	-864.081	1790.161
SYM	MaddFitz		-905.615	1841.230
ARD	MaddFitz		-902.593	1841.187
SYM	Yang		-907.049	1844.099
ARD	Yang		-904.480	1844.960
ER	MaddFitz	HMM	-993.637	1993.274
ER	Yang	HMM	-995.137	1996.275
ER	MaddFitz		-1027.774	2057.547
ER	Yang		-1029.081	2060.163

Supplementary Table 9: Log Marginal Likelihood (log ML) estimates using the standard versus covarion BayesTraits Multistate algorithms with reverse jump MCMC and a stepping stone sampler, for Anura, Caudata and Gymnophiona.

	Standard MultiState (log ML)	*Covarion MultiState (log ML)*	*Log BF*
Anura	-715.058	-714.497	1.123
Caudata	-132.682	-123.782	17.801
Gymnophiona	-38.285	-36.318	3.933

Supplementary Table 10: Posterior transition rate metrics for Anura, estimated using the covarion BayesTraits Multistate algorithms with reverse jump MCMC. Tabulated are the mean and median transition rates for each transition (where states are Aquatic [A], Semi-terrestrial [S], Terrestrial [T], Direct development [D] and Live-bearing [L]) as well as the percentage any given transition was not fixed to be zero in the posterior (i.e. how frequently is a given transition represented in the posterior) and the Effective Sample Size (ESS) + Highest probability Density interval (HPD) for the mean rate. Transitions are ranked by how frequently they were not zero.

transition	*mean*	*median*	*% non-zero*	*ESS*	*HPD interval*
qAD	0.955	0.908	100.000	2000.000	0.622,1.387
qAS	17.499	17.032	100.000	2000.000	8.756,26.956
qAT	0.955	0.909	100.000	2000.000	0.622,1.387
qDS	0.954	0.909	100.000	2000.000	0.622,1.387
qSA	0.948	0.908	100.000	2000.000	0.615,1.368
qST	0.948	0.908	100.000	2000.000	0.615,1.369
qAV	0.937	0.905	99.250	2000.000	0.616,1.4
qVA	0.937	0.905	99.250	2000.000	0.616,1.4
qTS	0.900	0.897	94.800	2000.000	0,1.331
qSD	0.760	0.848	85.200	2057.439	0,1.128
qTD	0.510	0.706	54.300	2000.000	0,1.219
qTA	0.461	0.000	48.750	2000.000	0,1.217
qTV	0.347	0.000	37.550	2000.000	0,1.15
qVT	0.347	0.000	37.550	2000.000	0,1.15
qDT	0.042	0.000	5.250	1581.571	0,0.593
qDA	0.034	0.000	4.100	2000.000	0,0
qDV	0.019	0.000	2.400	1254.320	0,0
qVD	0.019	0.000	2.400	1254.320	0,0
qSV	0.001	0.000	0.250	2000.000	0,0
qVS	0.001	0.000	0.250	2000.000	0,0

Supplementary Table 11: Posterior transition rate metrics for Caudata, estimated using the covarion BayesTraits Multistate algorithms with reverse jump MCMC. Tabulated are the mean and median transition rates for each transition (where states are Aquatic [A], Semi-terrestrial [S], Direct development [D], Live-bearing [L], and Paedomorphism[P]), as well as the percentage any given transition was not fixed to be zero in the posterior (i.e. how frequently is a given transition represented in the posterior) and the Effective Sample Size (ESS) + Highest probability Density interval (HPD) for the mean rate. Transitions are ranked by how frequently they were not zero.

transition	*mean*	*median*	*% non-zero*	*ESS*	*HPD interval*
qAP	6.862	6.367	100.000	2748.334	2.316,12.544
qAS	6.772	6.306	99.750	2693.498	2.282,12.85
qDS	5.281	5.273	89.825	2345.088	0,10.821
qSA	6.416	6.003	88.950	2450.807	0,12.556
qDA	4.748	5.005	80.125	2069.370	0,10.631
qSD	4.900	5.153	72.950	2283.220	0,11.501
qSL	3.841	3.959	64.650	1955.250	0,10.213
qLA	2.800	0.000	45.375	3324.537	0,9.972
qPA	2.318	0.000	44.675	3041.547	0,8.482
qLP	2.521	0.000	43.200	3282.483	0,9.444
qLS	2.584	0.000	43.200	2567.033	0,9.421
qLD	2.215	0.000	37.850	3527.581	0,9.315
qDP	1.908	0.000	36.075	2928.224	0,7.958
qSP	1.728	0.000	35.225	3922.913	0,8.111
qPD	1.581	0.000	35.050	2307.054	0,7.09
qPL	1.590	0.000	33.450	1817.466	0,7.603
qDL	1.333	0.000	25.050	2955.710	0,7.715
qPS	0.752	0.000	19.625	3347.350	0,5.555
qAL	0.424	0.000	18.900	2247.829	0,3.426
qAD	0.270	0.000	12.525	3029.397	0,1.546

Supplementary Table 12: Posterior transition rate metrics for Gymnophiona, estimated using the covarion BayesTraits Multistate algorithms with reverse jump MCMC. Tabulated are the mean and median transition rates for each transition (where states are Semi-terrestrial [S], Direct development [D] and Live-bearing [L]) as well as the percentage any given transition was not fixed to be zero in the posterior (i.e. how frequently is a given transition represented in the posterior) and the Effective Sample Size (ESS) + Highest probability Density interval (HPD) for the mean rate. Transitions are ranked by how frequently they were not zero.

transition	*mean*	*median*	*% non-zero*	*ESS*	*HPD interval*
qSD	7.570	5.528	95.280	13508.221	0,18.653
qDV	2.886	2.376	57.970	14402.577	0,7.887
qDS	2.920	2.357	57.815	14860.482	0,8.093
qSV	4.717	0.000	44.585	12816.359	0,17.582
qVD	2.989	0.000	41.820	17395.737	0,12.002
qVS	2.396	0.000	27.225	17852.140	0,12.107

Supplementary Notes 1: Root state estimations for Amphibia, using corHMM

Our hypothesis testing approach for reproductive mode evolution assumes that extant amphibians have evolved from fully aquatic ancestors. Various lines of evidence support this assumption (e.g. Hanken[1]; Schoch et al.[2]). To confirm nonetheless that our large phylogenetic dataset continues to recover aquatic biphasic reproduction as the ancestral condition, we estimated ancestral states for the entire amphibian tree (4025 species) using a Markov model implemented with corHMM[3] with and without "hidden" states, and two different root prior methods: MaddFitz[4,5] and Yang[6]. Ancestral states were estimated using marginal probabilities. All other settings are identical to those described in the main text.

As described in the methods of the main text (and shown in other studies, e.g. Davis et al.[7]) rare states (i.e. high tip state ratio bias) result in problematic parameter estimations. Specifically, we have found that in models with hidden states (where rare states become even rarer), transition rate estimates away from rare states were multiple order of magnitudes larger than any other rates. Three of our six reproductive-mode categories are rare. For the whole amphibian tree, the terrestrial mode is represented by 2.06%, paedomorphism by 0.86% and live bearing by 0.73% of species (compared to aquatic = 45.45%, semi-terrestrial = 23.47% and direct development =27.42%). Transitions to and away from these three rare states were therefore fixed to by symmetric for all twelve models, which we have found results in more credible transition rate estimates.

As expected, the most recent common ancestor for all amphibians, as well as for anurans and for caudates were estimated to have had an aquatic life history mode (Supplementary Figure 7, below). For Gymnophiona, we recovered a semi-terrestrial ancestor as the most likely scenario for Gymnophiona, in concordance with previous studies[8]. The best performing model was a model with no (other than the above mentioned) state transition restrictions (i.e. the ARD model), hidden states and a MaddFitz root prior (Supplementary Table 8, below) but the state estimations at the ancestral nodes were not sensitive to any of the tested model variations (Supplementary Figure 7).

References

1. Hanken, J. in *The Origin and Evolution of Larval Forms* 61–108 (Gulf Professional Publishing, 1999).
2. Schoch, R. Evolution of life cycles in early amphibians. *Annu. Rev. Earth Planet. Sci.* **37,** 135–162 (2009).
3. Beaulieu, J. M., Oliver, J. C., O'Meara, B. & Boyko, J. R package 'corHMM': hidden markov models of character evolution. (2020).
4. Maddison, W., Midford, P. & Otto, S. Estimating a binary character's effect on speciation and extinction. *Syst. Biol.* **56,** 701–710 (2007).
5. FitzJohn, R. G., Maddison, W. P. & Otto, S. P. Estimating trait-dependent speciation and extinction rates from incompletely resolved phylogenies. *Syst. Biol.* **58,** 595–611 (2009).
6. Yang, Z. *Computational Molecular Evolution*. (Oxford University Press, 2006). doi:10.1093/acprof:oso/9780198567028.001.0001
7. Davis, M. P., Midford, P. E. & Maddison, W. Exploring power and parameter estimation of the BiSSE method for analyzing species diversification. *BMC Evol. Biol.* **13,** 38 (2013).
8. San Mauro, D. *et al.* Life-history evolution and mitogenomic phylogeny of caecilian amphibians. *Mol. Phylogenet. Evol.* **73,** 177–189 (2014).
