# Supplementary material for: The evolution of reproductive modes and life cycles in amphibians
Source: Nat Commun. 2022 Nov 17;13:7039. doi: 10.1038/s41467-022-34474-4 (PMC9672123; doi:10.1038/s41467-022-34474-4)
Supplement: Supplementary file 2 — Description of Additional Supplementary Files [file 41467_2022_34474_MOESM2_ESM.pdf]

Description of Additional Supplementary Files

File Name: Supplementary Data 1
Description: Reproductive mode coding of amphibian species in this study.

File Name: Supplementary Data 2
Description: Rate estimates from best performing SSE models (both hisse and secsse)

File Name: Supplementary Data 3
Description: Taxonomic changes made to the phylogeny

File Name: Supplementary Data 4
Description: Phylogeny used for comparative analyses in this study.
