# Supplementary material for: The evolution of reproductive modes and life cycles in amphibians
Source: Nat Commun. 2022 Nov 17;13:7039. doi: 10.1038/s41467-022-34474-4 (PMC9672123; doi:10.1038/s41467-022-34474-4)
Supplement: Supplementary file 7 — Reporting Summary [file 41467_2022_34474_MOESM7_ESM.pdf]

nature portfolio

Corresponding author(s): H. Christoph Liedtke

Last updated by author(s): Oct 18, 2022

Reporting Summary

Nature Portfolio wishes to improve the reproducibility of the work that we publish. This form provides structure for consistency and transparency in reporting. For further information on Nature Portfolio policies, see our Editorial Policies and the Editorial Policy Checklist.

Statistics

For all statistical analyses, confirm that the following items are present in the figure legend, table legend, main text, or Methods section.

n/a	Confirmed	
☐	☒	The exact sample size (*n*) for each experimental group/condition, given as a discrete number and unit of measurement
☒	☐	A statement on whether measurements were taken from distinct samples or whether the same sample was measured repeatedly
☒	☐	The statistical test(s) used AND whether they are one- or two-sided<br>*Only common tests should be described solely by name; describe more complex techniques in the Methods section.*
☒	☐	A description of all covariates tested
☒	☐	A description of any assumptions or corrections, such as tests of normality and adjustment for multiple comparisons
☐	☒	A full description of the statistical parameters including central tendency (e.g. means) or other basic estimates (e.g. regression coefficient) AND variation (e.g. standard deviation) or associated estimates of uncertainty (e.g. confidence intervals)
☒	☐	For null hypothesis testing, the test statistic (e.g. *F*, *t*, *r*) with confidence intervals, effect sizes, degrees of freedom and *P* value noted<br>*Give P values as exact values whenever suitable.*
☐	☒	For Bayesian analysis, information on the choice of priors and Markov chain Monte Carlo settings
☒	☐	For hierarchical and complex designs, identification of the appropriate level for tests and full reporting of outcomes
☒	☐	Estimates of effect sizes (e.g. Cohen's *d*, Pearson's *r*), indicating how they were calculated

Our web collection on statistics for biologists contains articles on many of the points above.

Software and code

Policy information about availability of computer code

Data collection	AmphiNom v1.0.1
Data analysis	R v3.6.1; corHMM v2.3; hisse v 1.9.6; secsse v2.1.7; AmphiNom v1.0.1; DDD v5.0; BayesTraits v4; Custom code is available at DOI: 10.5281/zenodo.7215080

For manuscripts utilizing custom algorithms or software that are central to the research but not yet described in published literature, software must be made available to editors and reviewers. We strongly encourage code deposition in a community repository (e.g. GitHub). See the Nature Portfolio guidelines for submitting code & software for further information.

Data

Policy information about availability of data

All manuscripts must include a data availability statement. This statement should provide the following information, where applicable:

- Accession codes, unique identifiers, or web links for publicly available datasets
- A description of any restrictions on data availability
- For clinical datasets or third party data, please ensure that the statement adheres to our policy

Supplementary Data 1 contains the reproductive mode data for each species and supporting references. Supplementary Data 2 rate estimates for the best peforming hisse and secsse models. Supplementary Data 3 contains the taxonomic changes made to the phylogeny. Supplenetary Data 4 contains the phylogeny used for the comparative analyses. The taxonomy used follows that of the Amphibian Species of the World v6.0 (https://amphibiansoftheworld.amnh.org/). Source data are provided with this paper.

Field-specific reporting

Please select the one below that is the best fit for your research. If you are not sure, read the appropriate sections before making your selection.

☐ Life sciences ☐ Behavioural & social sciences ☒ Ecological, evolutionary & environmental sciences

For a reference copy of the document with all sections, see nature.com/documents/nr-reporting-summary-flat.pdf

Ecological, evolutionary & environmental sciences study design

All studies must disclose on these points even when the disclosure is negative.

Study description	The study tests statistical associations between categorical amphibian life-history modes and aspects of evolutionary dynamics such as lineage diversification rates and trait transition rates. No experiment is performed.
Research sample	Reproductive modes for 7681 species of amphibians (spanning 3 orders and 75 families) were coded. This is approximately 95% of all amphibian species. This dataset was matched to a modified phylogeny published by Jetz, W. & Pyron, R.A. (2018). Nature Ecology & Evolution, 2, 850-858, representing 4025 species in our reproductive mode dataset.
Sampling strategy	We targeted all amphibian species: 8,047 at the time of data collection (following: https://amphibiansoftheworld.amnh.org/). For 366 species, no reliable data could be found.
Data collection	Life-history mode categorization is based on where and how eggs are deposited and where and how larva develop. This information was compiled manually by the authors of this study, by screening online resources including AmphibiaWeb and the IUCN Red List. All sources are listed in the supplementary data 1 file.
Timing and spatial scale	The data was collected in August 2019, with a review and minor updates in December 2020 to incorporate newly published data.
Data exclusions	No data was excluded.
Reproducibility	Analyses involving likelihood searches (corHMM, hisse and secsse) were repeated up to 25 times with different starting parameters (see R code made available with this study for details) to ensure arriving at the global maximum. The Bayesian analysis (BayesTraits) was repeated three times to ensure chain convergence. All findings can be reproduced in full with the code made available.
Randomization	This study contains no experimental component where randomization would be applicable.
Blinding	This study contains no experimental component where blinding would be applicable.

Did the study involve field work? ☐ Yes ☒ No

Reporting for specific materials, systems and methods

We require information from authors about some types of materials, experimental systems and methods used in many studies. Here, indicate whether each material, system or method listed is relevant to your study. If you are not sure if a list item applies to your research, read the appropriate section before selecting a response.

Materials & experimental systems

n/a	Involved in the study
☒	☐ Antibodies
☒	☐ Eukaryotic cell lines
☒	☐ Palaeontology and archaeology
☒	☐ Animals and other organisms
☒	☐ Human research participants
☒	☐ Clinical data
☒	☐ Dual use research of concern

Methods

n/a	Involved in the study
☒	☐ ChIP-seq
☒	☐ Flow cytometry
☒	☐ MRI-based neuroimaging
